# Transcriptional, Epigenetic and Metabolic Programming of Tumor-Associated Macrophages

**DOI:** 10.3390/cancers12061411

**Published:** 2020-05-29

**Authors:** Irina Larionova, Elena Kazakova, Marina Patysheva, Julia Kzhyshkowska

**Affiliations:** 1 Laboratory of Translational Cellular and Molecular Biomedicine, National Research Tomsk State University, 634050 Tomsk, Russia; larionova0903irina@mail.ru (I.L.); kazakova.e.o@mail.ru (E.K.); starin5@yandex.ru (M.P.); 2 Cancer Research Institute, Tomsk National Research Medical Center of the Russian Academy of Sciences, 634009 Tomsk, Russia; 3 Institute of Transfusion Medicine and Immunology, Medical Faculty Mannheim, University of Heidelberg, 68167 Mannheim, Germany; 4 German Red Cross Blood Service Baden-Württemberg—Hessen, 68167 Mannheim, Germany

**Keywords:** tumor-associated macrophages, reprogramming, tumor, metabolism, glycolysis, fatty acid oxidation, epigenetic regulation, histones, methylation, transcription factors

## Abstract

Macrophages are key innate immune cells in the tumor microenvironment (TME) that regulate primary tumor growth, vascularization, metastatic spread and tumor response to various types of therapies. The present review highlights the mechanisms of macrophage programming in tumor microenvironments that act on the transcriptional, epigenetic and metabolic levels. We summarize the latest knowledge on the types of transcriptional factors and epigenetic enzymes that control the direction of macrophage functional polarization and their pro- and anti-tumor activities. We also focus on the major types of metabolic programs of macrophages (glycolysis and fatty acid oxidation), and their interaction with cancer cells and complex TME. We have discussed how the regulation of macrophage polarization on the transcriptional, epigenetic and metabolic levels can be used for the efficient therapeutic manipulation of macrophage functions in cancer.

## 1. Introduction

Tumor microenvironment (TME) is the place of intimate crosstalk between all cellular components, including malignant, endothelial, stromal, and immune cells [1]. The TME shapes the intracellular program of cells, regulating their functionality. Signals that are involved in such regulations are cytokines, growth and transcription factors, oxygen levels, and nutrients [1,2]. Immune cells in the TME reprogram their phenotype to a tumor-associated one, maintaining the survival, growth, and proliferation of tumor cells [3]. In this context tumor-associated macrophages (TAMs) are one of the main cellular components involved in tumor progression, by regulating angiogenesis, initiation and growth of tumors, angiogenesis, lymphangiogenesis, local and distant metastasis [4,5,6]. Macrophages are extremely plastic cells that respond to stimuli from the local microenvironment acquiring a specific phenotype and reflecting the functionally distinct macrophage populations [7].

Macrophages can be classified into two major subtypes that reflect two major vectors of functional polarization: classically activated pro-inflammatory, or M1 macrophages, and alternatively activated anti-inflammatory, or M2 macrophages [8,9]. However, this nomenclature is artificial and reflects in vitro generated subtypes, while macrophages in vivo (including TAMs) are highly diverse cells, and can combine M1 and M2 molecular characteristics and functions. A number of studies have shown that changes in metabolism, transcriptome, and epigenetic-associated mechanisms provide macrophages with unique functional plasticity that is detrimental when they respond to cancer cell-derived signals and start to support tumor progression. However, such plasticity makes TAMs highly attractive targets for therapeutic reprogramming. Complex interaction in TME often involves extracellular metabolites that act as communication signals [2,10]. By changing the metabolism and transcriptome of macrophages, it will be possible to modulate their functions making them beneficial for the treatment of patients with cancer. For example, depending on the stimuli, macrophages can switch from the oxidative phosphorylation to the glycolysis, and vice versa [10,11]. Recent studies have shown a number of transcriptional factors participating in the differential activation of macrophages [12,13]. A class of small noncoding RNAs, microRNAs, also were found to participate in macrophage polarization [14]. Moreover, programs for the differentiation of monocytes and maturate macrophages are based on the significant epigenetic modifications (DNA methylation, histone modifications, miRNA.) [15].

In this review, we focus on three major mechanistic levels that define macrophages phenotype and functional polarization: transcriptional factors, epigenetic modifications, and metabolic pathways. We discuss these three mechanistic levels in the context of programming of macrophage functions in cancer, and outline the perspectives for the reprogramming of TAMs to develop complex and personalized anti-cancer therapeutic approaches.

## 2. Transcription Regulation of Macrophage Polarization

**Figure 1:**
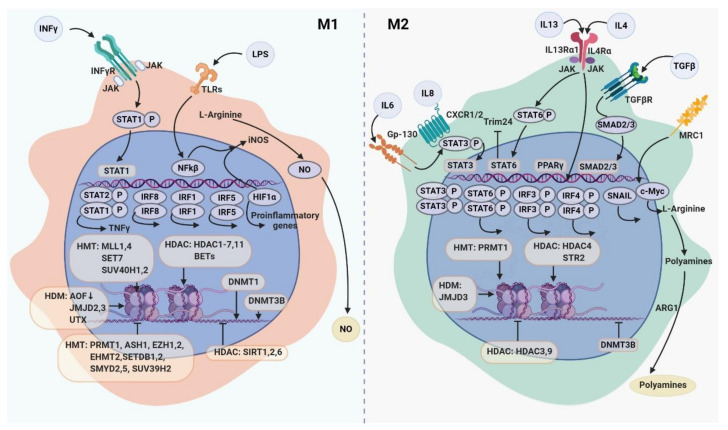
Transcription factors and epigenetic enzymes involved in macrophage polarization. ARG1—Arginase 1; HDAC—Histone deacetylase; HDM—Histone demethylase; HMT—Histone methyltransferase; P—phosphorylated form. Figure created in biorender (http://biorender.io).

Transcription factors (TFs) respond to virtually all stimuli of the tumor microenvironment including cytokines, growth factors, extracellular matrix (ECM) components, metabolic factors, and control gene expression through the transactivation or transrepression domains [16]. TF activity is mediated by complex of functional domains, through which TFs binds to the appropriate DNA strand, interacts with other TFs, coactivators, and RNAII polymerase enzyme [17], chromatin remodeling complexes, and small noncoding RNAs [14]. More than half of known TFs in the genome are expressed in macrophages under the different states of polarization, and functional activation of macrophages is controlled by number of TFs [2,18]. Below we summarize the knowledge about major transcription factors that define development, activation and plasticity of macrophages in the context of the TME (Table 1, Figure 1).

### 2.1. PU.1

PU.1 is a prominent transcriptional regulator of myeloid cell development and phenotype plasticity [19,63,64]. It is a principal transcription factor that activates promoters of Csf1r gene encoding a key receptor for the macrophage lineage commitment and regulation of macrophage, differentiation and functional activation [65]. Both interferon (IFN) regulatory factors IRF8 and IRF4 bind PU.1 cooperatively at the IRF/PU.1 site in RAW264.7 cells [66]. PU.1 promotes macrophage differentiation toward alternatively activated macrophages and is involved in the development of many types of tumors including breast cancer [67], myeloma [68], acute myeloid leukemia [69], glioma [70] and hepatocellular carcinoma [71].

PU.1 mediates monocyte/macrophage differentiation via activation of miR-22 in human leukemia cell line (HL-60), human monocytic cell line (THP1) cells and CD34+ hematopoietic stem/progenitor cells [72]. In vitro, PU.1 was found to be a critical regulator of M2 polarization via the IL-4/STAT6 signaling pathway in murine bone marrow-derived macrophages (BMDMs) [48] (Table 1). PU.1-deficient murine macrophages displayed decreased expression of IL-4-induced specific markers, chitinase 3-like 3 (Ym-1) and resistin-like molecule alpha 1 (Fizz-1) [48]. PU.1 knockdown resulted in reduced alternative activation of macrophages that was associated with decreased expression of CCL22, while lipopolysaccharide (LPS) treatment resulted in up-regulation of PU.1 expression accompanied by increased level of CCL22 in murine BMDMs [49]. There is also evidence about the involvement of PU.1 in the regulation of M1 polarization. Thus, miR-181a induces macrophage polarization to M2 phenotype through suppression of the expression of PU.1, C/EBPα and KLF6 in human macrophages [50]. PU.1 is a transcription factor required for the efficient inflammatory reactions in macrophages. Thus, in a mouse model with functional PU.1 knockout in mature macrophages, the inhibition of inflammatory gene expression (COX-2, iNOS, TLR4) and inflammatory cytokine secretion (IL-6, MCP-1, IL-1β, TNF-α), as well as significant decrease in systemic inflammation, was identified [51]. Although during the last decade significant progress in the study of PU.1-mediated plasticity of macrophages was achieved, the mechanism of PU.1-shaped phenotypes of macrophages in the tumor microenvironment remains to be incompletely understood.

### 2.2. STAT Family

Signal transducers and activators of transcription (STATs) are a family of transcription factors that were originally identified as classic effectors of interferon-induced signaling. STATs affect macrophage phenotypes in response to cytokines and growth factors through the different signaling pathways underlying the role of STATs on TAM functional programming [26,51,54,55,57,58,59,60,61,73,74,75,76,77] (Figure 1). Thus, STAT1 mediates M1 macrophage polarization via the IFNγ and TLR signaling pathways [54]. In patients with locally advanced cervical cancer the increase in the amount of CD68+pSTAT1+ cells, defined as M1 macrophages, in tumor mass was associated with a longer disease-free survival (DFS) and overall survival (OS) [55]. However, in contrast to human studies, STAT1, but not STAT3 or STAT6, was responsible for immunosuppressive activity of TAMs derived from colon CT-26 tumor-bearing BALB/c mice [56].

STAT3 is involved in angiogenesis and tumor progression through polarization of TAMs to the M2 phenotype [57,73]. In RAW264.7 cells (mouse macrophage cell line) and in BMDMs, STAT3 phosphorylation mediates IL-4 and TGFβ1-induced macrophage polarization toward the M2 phenotype that is exacerbated by Wnt3a [58]. In a co-culture of hepatocellular carcinoma (HCC) cells and macrophages, IL-6/STAT3 signaling pathway was suppressed in M1 macrophages but was activated in M2 macrophages [59]. Similar result was obtained in monocytes of healthy donors cultivated in the presence of PC3 (prostate cancer cell line) conditioned medium where M2 phenotype was characterized by IL-10-induced phosphorylation of STAT3 [57] (Table 1). In tumor-associated myeloid-derived suppressor cells (MDSCs), STAT3 was required for the induction of angiogenic factors, including VEGF and bFGF, and increased angiogenesis in vitro [60]. Phosphorylated STAT3 and STAT6 together cooperated to increase cathepsin expression in TAMs resulting in the enhanced tumor invasion in vivo [74]. STAT6 mediates the stimulation of M2-like polarization of macrophages in response to IL-4 and/or IL-13, mediators of Th2 immune responses [75] (Figure 1). IL-4-driven activation of STAT6 leads to the inhibition of TRIM24 activity in macrophages, supporting polarization of macrophages toward the tumor-associated phenotype in a murine model of melanoma [61]. In a murine model of colorectal cancer, activated STAT6 and KLF4 are involved in MFHAS1-induced M2 polarization of TAMs leading to tumor progression [76]. In murine mammary carcinoma, TAMs facilitate metastatic colonization by secretion of IL-35 through activation of JAK2–STAT6-GATA3 signaling [77].

There are several therapeutic approaches suggested for the inhibition of tumorigenic action of STATs in macrophages. For example, liposome-encapsulated STAT3 inhibitor can activate reprogramming of CD163+TAMs toward pro-inflammatory phenotypes with increased secretion of IFNγ, IL-12, TNFα, IL-2 in vitro [78]. Another study demonstrated that herbal acidic polysaccharide IAPS-2 inhibits the phosphorylation of STAT3 and enhances STAT1 phosphorylation in TAMs from S180 tumor tissues (a syngeneic sarcoma) promoting macrophage polarization toward the M1-like phenotype [79]. Inhibition of the STAT6 pathway by using small interfering RNA or the pharmacologic inhibitor AS1517499 inhibited the differentiation of murine RAW264.7 macrophages into the M2 phenotype, as demonstrated by the reduction of ARG1 and CD206 expression [77]. Besides, AS1517499 significantly attenuated tumor growth and early liver metastasis in 4T1 mammary carcinoma mouse model [77].

Thus, transcription factors from STAT family are involved in the macrophage plasticity by programming phenotypes towards M1 or M2 in response to the temporal and spatial stimuli in the tumor microenvironment (Table 1).

### 2.3. NF-kB

Transcription factors of nuclear factor-κB (NF-κB) family regulate the expression of genes that control inflammation, immune responses, cell survival, cell proliferation and differentiation [80]. Inflammation has a dual role in cancer progression [81]. Inflammation in the microenvironment supports cell transformation and intratumoral mutagenesis [82]. On the other hand, induction of inflammation may have a potent anti-tumor effect. NF-κB is a key transcription factor of M1 polarization which is required for induction of a number of pro-inflammatory cytokines [46,47,83]. Thus, RAW 264.7 cells stimulated by IFN-γ are polarized to M1 macrophages via NF-κB signaling pathway [84]. In the tumor microenvironment, TAM-derived IL-10 inhibits IL-12 production associated with the lack of NF-κB activation promoting tumor survival, while blocking of IL-10 restores the IL-12 production in a mouse model of fibrosarcoma [85] Tumor-promoting activation of NF-κB in macrophages was also demonstrated [46]. TAMs polarized to immunosuppressive phenotype with high expression of IL-10, TNF-α, and ARG1, but low expression of NOS2, IL-12, and MHC II, that was mediated by the IL-1R and MyD88 via NF-κB activation, resulted in increased tumor invasiveness and tumor growth in ovarian cancer in vitro and in vivo [46]. IL-17 promotes THP-1 cell differentiation towards M2-like phenotype (characterized by increased expression of CD163 and CD206, TGF-β, VEGF and IL-10 production) through NF-κB signaling pathway [47] (Table 1). Another study showed that expression of PD-1 in RAW264.7 cells can be regulated by TLR/NF-κB signaling [83].

Despite the fact that NF-κB is considered as a potential activator of pro-inflammatory M1 phenotype, it seems that the role of NF-κB signaling in TAM plasticity depends on the stimuli from the TME and from the type of cancer.

### 2.4. c-Myc

c-Myc was identified in 1981 as a gene activated by avian leukosis virus that was implicated in the development of bursal lymphomas [86]. c-Myc is a member of the Myc family of transcription factors that regulate broad range of cellular processes including cell cycle, metabolism, epithelial–mesenchymal transition (EMT), metastasis and angiogenesis, thereby playing a crucial role in genesis of tumor disease and tumor progression [87]. c-Myc was identified as M2-polarizing transcription factor in murine macrophages [20]. Transcriptomic analysis of murine BMDMs demonstrated that c-Myc is a marker of M2 macrophages activated by IL-4 [20]. c-Myc modulates M2-polarization via IL-4–dependent induction of genes involved in alternative activation of human macrophages (e.g., SCARB1, ALOX15, and CD206) [21]. c-Myc inhibition by treatment with 10058-F4 or transduction of c-Myc by c-Myc/shRNA in human macrophages stimulated by tumor-conditioned medium from PANC-1 (human pancreatic cancer cell line) suppresses expression of protumoral genes (ALOX15, CD206, TGF-β, VEGF, HIF-1α and MMP9) [21] (Table 1). c-Myc is expressed in CD68+ TAMs [21]. STAT6 is required for c-Myc modulated alternative type of macrophage polarization [88]. The recent study in mature murine BMDMs cultured in conditioned medium of Hepa1-6 (murine hepatoma cells) demonstrated that Wnt/β-catenin signaling mediates polarization of M2 macrophages through activation of c-Myc that supports the progression of hepatocellular carcinoma (HCC) [22]. Interestingly, in the co-culture model of human monocytes and HCC cells, IL-12 inhibits c-Myc and STAT3 transcription factors in monocytes, mediates M1 polarization and suppresses the HCC growth [89]. Moreover, deletion of c-Myc in macrophages resulted in the reduced expression of pro-tumor genes (e.g., VEGF, MMP9, and HIF1a) in TAMs and reduced tumor development in a mouse model of melanoma [23]. Thus, c-Myc is an essential transcription factor that defines development of pro-tumoral phenotype of TAMs.

### 2.5. Interferon Regulatory Factors

Other transcription factors involved in the polarization of macrophages include a family of interferon regulatory factors (IRFs) that have been originally identified as transcription activators and repressors of interferon (Table 1, Figure 1). The family of IRFs includes nine members: IRF1, IRF2, IRF3, IRF4/PIP/LSIRF/ICSAT, IRF5, IRF6, IRF7, IRF8/ICSBP, and IRF9/ISGF3γ, that participate in the regulation of both development and activation of the immune system cells [90]. Notably, IRF1, IRF5, and IRF8 contribute to pro-inflammatory polarization of macrophages while IRF3 and IRF4 regulate M2 polarization macrophages [24,25,31,33]. Thus, IRF1 is involved in M1 polarization in human macrophage cell line U937 in response to IFNγ and LPS by upregulation of IL-12, IL-6, IL-23 and CD86 and downregulation of M2-specific marker CD206 [24]. The knockdown of IRF1 in macrophages induces their pro-tumor activity regarding to hepatocellular carcinoma cell lines HepG2 and SMMC-7721, promoting proliferation and invasion of tumor cells [24].

IRF3 promotes M-CSF-mediated differentiation of monocytes toward M2 type macrophages [25]. IRF3 activates PI3K/Akt signaling mediating the inhibition of pro-inflammatory genes (IL-1α, IL-1β, TNFα, IL-6, IL-8, and CXCL1) and stimulation of anti-inflammatory genes (IL-1RN, IL-10, and IFN-β) in human fetal microglia [26]. Under LPS treatment, TAMs isolated out of murine fibrosarcoma showed impaired MyD88-dependent NF-κB activation and activation of the MyD88-independent IRF-3 pathway [27]. This was consistent with low expression of several pro-inflammatory cytokines (e.g., IL-1β, IL-6, TNF-α) and up-regulation of immunosuppressive cytokines (IL-10, TGFβ) and IFN-inducible chemokines (CCL5, CXCL9, CXCL10, and CXCL16) [27]. In a murine model of nasopharyngeal carcinoma, cancer progression is mediated by EBV encoded RNAs (EBER)-triggered inflammation dependent on the phosphorylation of p38 and IRF3 [91]. However, TAM polarization to pro-inflammatory M1 status can also dependent on TLR3- and TLR4-IRF3 signaling [28,29]. IRF3 phosphorylation and transcriptional activity is regulated by Smad2 and Smad3 [92]. Double knockdown of Smad2\3 in BMDMs is critical for the phosphorylation of IRF3 and STAT1 transcriptional activities and IFN-β production in response to LPS [92]. Another study has demonstrated the inhibition of pro-tumorigenic genes encoding VEGF and MMP2 in IRF3- and IRF7-transduced macrophages. Additionally, IRF7 displayed cytotoxic activity of macrophages in breast cancer (SK-BR-3, MCF-7) and colorectal cancer (COLO-205) cell lines [30]. IRF7 also can serve as a factor, regulating IL-10 response [35]. In human monocyte-derived macrophages IRF-7 knockdown by siRNA increased LPS-induced IL-10 production, indicating that IRF7 induction blocks early IL-10 response [35].

IRF4 and histone demethylase Jumonji domains containing-3 (Jmjd3) are important players in IL-4-induced M2 polarization of macrophages acting through the activation of M2-specific genes (ARG1, FIZZ1, Ym1, and CD206) [31]. The level of IRF4 protein together with STAT3 and P-STAT3 proteins was elevated in monocyte-derived M2 macrophages induced by IL-6 in vitro [32]. ChIP assay demonstrated that IRF4 can be recruited to the PU.1 site and trans-activated by the MR enhancer reporter (pGL3-MR) in RAW264.7 cells, while transfection of macrophages with miR-125a suppressed IRF4 expression and pGL3-MR transactivation [93]. Interestingly, the miR-23a/27a/24-2 cluster reduced the production of M2 type cytokines by directly targeting JAK1/STAT-6 pathway with miR-23a and by targeting IRF4 and PPAR-γ with miR-27a [94]. qRT-PCR analysis of tumor samples from renal cell carcinoma patients revealed positive correlation between M2-associated genes (CD163, FN1 and IRF4) and reduced survival [95].

High expression of IRF5, in contrast, is associated with activation of inflammatory gene expression (IL-12p40, IL-12p35 and IL-23p19) and inhibition of anti-inflammatory genes that promotes M1 polarization in macrophages [34]. Furthermore, co-expression of IRF5 and IKKβ (a kinase that phosphorylates and activates IRF5) mediates TAM polarization towards M1 phenotype, supressing tumor development in model systems of advanced-stage ovarian cancer, metastatic melanoma, and glioblastoma [33].

Another study demonstrated that Notch-RBP-J signaling regulates expression of IRF8 inducing the expression of M1-specific genes in RBP-J deficient mice [18]. Moreover, IFN-γ-induced IRF8 is involved in the activation of transcription of pro-inflammatory genes [96]. Inhibition of IRF8 in macrophages reduces expression of inflammatory mediators associated with M1 macrophage (IL-1b, IL-6, iNOS, and TNF-α) and delayed wound healing in vivo [97]. IRF8 deficiency in macrophages significantly increased metastasis and expression of metastatic-associated genes in the mouse models of mammary cancer and melanoma, and correlated with reduced survival in human breast and lung cancers and melanoma [36]. High levels of IRF8 expression is associated with prolonged DFS in renal cell carcinoma patients [37].

Thus, IRFs play an essential role both in the polarization of macrophages and in the formation of tumor-associated phenotype. The direction of the pro- or anti-tumoral effects depends on the type of IRF.

### 2.6. SNAIL Family

The regulatory role of macrophage polarization was also found for SNAIL family members, differentially expressed both in TAMs and in cancer cells [98]. This family consists out of three members: SNAIL1 (SNAIL), SNAIL2 (SLUG) and SNAIL3 (SMUC) that contain a zinc finger-binding domain. Transcriptional regulation by SNAIL has been involved in various biological processes in cells, including modulation of EMT via the inhibiting E-cadherin transcription, and regulation of cell adhesion [99]. In THP-1 cells, SNAIL participates in TGF-β induced activation of M2-like phenotype through the PI3K/AKT and Smad2/3 signaling pathways [53] (Table 1). At the same time, M1 polarized macrophages displayed reduced expression of lysine-specific demethylase 1 (LSD1) and SNAIL. The LSD1 inhibitor phenelzine increased expression of M1-like signatures both in vitro and in vivo in a murine model of triple-negative breast cancer [100]. Overexpression of SNAIL in human head and neck cancer cells regulates the transcription of microRNA-21 that promotes the production of miR-21-containing exosomes from tumor cells [98]. When CD14+ monocytes engulf tumor-derived miR-21-containing exosomes, they display increased expression of M2-like markers (CD206, CD163, IL-10) and down-regulation of M1-like markers (IL-18, IL-12B, HLA-DR). Knockdown of miR-21 in cancer cells attenuated the SNAIL-mediated M2 polarization, angiogenesis, and tumor growth [98].

### 2.7. Maf

Maf family of transcription factors comprises MafA, MafB, Maf (also known as c-Maf), NRL11, MafF12, MafG13 and MafK. Maf family belongs to the AP-1-type superfamily bZip and participates in the proliferation and differentiation of hematopoietic cells [101]. MafB (v-maf musculo-aponeurotic fibrosarcoma oncogene homolog B) and c-Maf are well-known transcription regulators of macrophage differentiation and polarization in both human and murine models [43,102,103,104]. In BMDMs from adult wild-type mice the expression of MafB was induced by IL-10 or IL-4/IL-13 and suppressed by LPS or GM-CSF. In the same model, c-Maf expression was induced by IL-10 and suppressed by IL-4/IL-13 or GM-CSF [102] (Table 1). MafB induced by IL-10 in human primary macrophages activated STAT3 signaling pathway leading to the increased expression of MMP9 and IL-7R genes [42]. LPS-stimulated peritoneal macrophages derived from macrophage-specific dominant-negative MafB transgenic mice showed increased expression of IL-6 and TNF-a [104]. MafB+ macrophages expressed high levels of IL-10, ARG1 and TNF-α in Lewis lung carcinoma (LLC) of MafB-GFP knock-in heterozygous mice [43]. Besides, strong expression of MafB was identified by immunostaining analysis in CD204+ and CD68+ TAMs on stage 3 of human lung cancer [43]. Elevated expression of MafB in TAMs was also demonstrated in a mouse model of breast cancer [105]. A recent study found that M2 macrophages induced by IL-4 and IL-13 express high levels of c-Maf that regulates expression of M2-related genes (IL-12, IL-1b, IL-6, ARG1, IL-10, VEGF, TGFb, IRF4, and CCR2) [106]. c-Maf is expressed by TAMs in human non-small cell lung carcinoma (NSCLC), and promotes M2-mediated T cell suppression and tumor progression by controlling M2-related genes in vivo [106]. Deletion of c-Maf in macrophages resulted in reduced tumor size and enhanced antitumor T cell immunity in vivo [106]. Thus, the tumor-supporting role of Maf in TAMs was found in several cancer models in mice as well as in human tumors.

### 2.8. Other Transcription Factors Involved in TAM Polarization

The Microphthalmia family of bHLH-LZ transcription factors (MiT/TFE) is a family of four leucine zipper transcription factors: MITF, TFEB, TFE3 and TFEC [107]. The MiT family members are involved in many basic cellular processes including lysosomal biogenesis and autophagy [108]. MITF family members are expressed in macrophages, and TFEC is a macrophage-specific transcription factor [109]. TFEB regulates TAM polarization in the tumor microenvironment. Knockdown of TFEB with TFEB shRNA lentivirul vector in mouse peritoneal macrophages resulted in the suppression of expression of M1 markers (NOS and TNF-α) and stimulation of expression of M2 markers (ARG1 and YM-1) [62]. In co-culture experiment of breast cancer cell line and macrophages, TFEB-knockdown in macrophages promoted their polarization to the M2-like phenotype through the downregulation of SOCS3 production and STAT3 activation. TFEB knockdown in EO771 or LLC-derived C57BL/6 mice resulting in enhanced angiogenesis, tumor growth and reduced infiltration of CD8+ T cells [62]. Besides, the activation of TFEB by hydroxypropyl-β-cyclodextrin in macrophages suppressed their M2 polarization and inhibited breast tumor growth in mice [62].

Kruppel-like factors (KLF) family is comprised of 17 zinc-finger transcription factors [110]. KLF4 and KLF6 regulate key cellular processes, such as differentiation, proliferation, and programmed cell death [38,40,41,111]. KLF4 induces M2-like polarization via STAT6 signaling and reduces M1-like activation depending on NF-κB activation in RAW264.7 cells [38]. In murine peritoneal macrophages, KLF4 and STAT6, induced by IL-4, promoted M2 polarization of macrophages via MCPIP (monocyte chemotactic protein-induced protein) activation and up-regulation of expression of ARG1 and FIZZ1 [39]. KLF4 and MCPIP suppressed LPS-induced expression of NF-κB target genes (iNOS, IL-1β, TNFα and IL-6) and inhibited M1 polarization [39]. Deletion of KLF4 in murine myeloid cells resulted in suppression of expression of M2 markers (ArRG1, CD206, IL-10, TGF-β1, and Chil3) and reduction of HCC growth [112]. KLF4 stimulates M2 polarization of TAMs via Hedgehog signaling pathway in LLC1-derived mice [112].

KLF6 is required for LPS and IFN-γ-induced macrophage polarization to M1-like phenotype acting in cooperation with NF-kB signaling [40]. It inhibits anti-inflammatory gene expression by downregulating PPARγ expression in macrophages (RAW264.7 cells and BMDMs) in vitro [40]. KLF6 mediates activation of pro-inflammatory gene signature through activation of NFκB signaling, and inhibits anti-inflammatory gene expression through the downregulation of STAT3 signaling in vitro in RAW264.7 cells and in vivo in KLF6-KO mice [41].

Transcription factor NFAT5 drives pro-inflammatory activation of both M1 (activating IL-12) and M2 (activating FIZZ-1 and ARG1) macrophages [45]. NFAT5-deficient macrophages had reduced pro-inflammatory status, followed by the reduced infiltration of cytotoxic CD8+ T cells into the tumor and the enhanced tumor growth of LLC and ID8 ovarian carcinoma models [45].

Thus, we can conclude that the polarization of macrophages toward pro-inflammatory or anti-inflammatory phenotypes depends on the variety of transcription factors. At the same time, TFs are activated by different signals from the microenvironment resulting in functional reprogramming of macrophages. Targeting of transcription factors in macrophages is a promising strategy to use macrophage plasticity for the reprogramming TAMs by blocking their tumor supporting activity and by activating their intrinsic anti-tumor functions (recognition and killing of transformed cells). However, specific delivery of the inhibitors to TAMs avoiding other cell types in various organs is still a biotechnological challenge.

## 3. Epigenetic Regulation of TAMs

The epigenetic level of regulation is critical for the differentiation and functional programming of macrophages [15,113]. There are three levels of epigenetic control of macrophages differentiation and activation: DNA methylation, histone modifications, and microRNA [15,114]. DNA methylation is essential for the macrophage differentiation [115,116]. Histone methylation is a principal epigenetic mechanism for activation of inflammatory reactions in macrophages. The regulatory role of epigenetic remodeling by microRNA has been observed in differentiation and functional activation of macrophages [117,118,119]. Epigenetic differences between M1 and M2 macrophages act as important functional determinants [15,120,121].

### 3.1. DNA Methylation

DNA methylation is methylation of 5′-carbon on cytosine bases located frequently in CpG islands of promoters [122,123,124]. DNA methylation prevents transcriptional machinery from the assembling on the altered promoter that leads to the silencing of gene transcription [122]. There are two states of DNA methylation: hypermethylation (gain–CH3) and hypomethylation (loss–CH3). Hypermethylation is characterized by the transfer of a methyl group to the cytosine ring in DNA by DNA methyltransferases (DNMTs) to form 5-methylcytosine. DNMT1, DNMT-3A and DNMT-3B are involved in this reaction [125,126]. Hypomethylation is a removal of methyl groups by ten-eleven translocation (TET) proteins [127,128]. DNA methylation in CpG islands is an active mechanism of the repression of gene expression [129,130,131]. Moreover, CpG methylation prevents also aberrant intragenic transcriptional initiation [130,131]. In cancer, DNA methylation is critical for the suppression of the expression of tumor suppressor genes while loss of DNA methylation leads to the overexpression of oncogenes.

There are evidences that DNA methyltransferases have specific effect on the formation of macrophage phenotypes (Figure 1). DNMT3b knockdown promotes macrophage polarization to alternatively activated M2 phenotype in RAW264.7 cells [132]. DNMT1 is implicated in M1 polarization by silencing the SOCS1 gene and a subsequent increase in TNF and IL-6 production [133]. Overexpression of DNMT1 promotes LPS- and IFN-γ-induced M1 activation whereas inhibition of DNMT1 attenuates it [133] (Table 2). Upregulation of DNMT1 correlates with decrease in peroxisome proliferator-activated receptor gamma (PPAR-γ) and with the increased production of pro-inflammatory cytokines in peripheral blood monocytes isolated from patients with atherosclerosis and in macrophages from adipose tissue [116,134]. In type 2 diabetic mice, decrease in the ability of macrophages to support wound healing was associated with microRNA let-7d-3p, which was up-regulated by DNMT1 resulting in the differentiation of cells toward the M1 phenotype [116]. However, the effect of LPS in BMDMs during M1 activation is also associated with a significant reduction in the expression of DNMT 1, 3a and 3b, and a significant increase in the expression of TET2 and TET3 [116] (Table 2, Figure 1). TET2 expression is increased in intratumoral myeloid cells, both in a mouse model of melanoma and in melanoma patients, that is dependent on an IL-1R-MyD88 pathway [135]. Recently, the combination of mass spectrometry and single molecular imaging demonstrated that LPS induces global changes in DNA methylation of the genome of murine macrophages [113,134].

Despite the clearly established role of DNA methylation in the classical inflammatory macrophage models, its role in the formation of TAM phenotypes in various tumor types is not understood. There are only some isolated reports showing that DNA methylation is involved in the MDSC function [176]. Since DNA methylation is a critical factor for cancer cell biology, there are a number of studies trying to identify epigenetic enzymes as targets for anti-cancer therapy. Therefore, understanding of the mechanism and functional consequences in DNA methylation in TAMs is urgently needed.

### 3.2. Histone Modification

Histone modifications, also known as histone code, provide a highly flexible mechanism for activation and deactivation of transcription in macrophages in response to the changing context of stimuli in the TME. Histone modifications in various cell types include a number of post-translational modifications such as methylation, acethylation, ubiquitination, arginine citrullination, sumoylation. The histone code is an essential mechanism that controls the activity of cancer cells [177]. The most frequent histone modifications, also found in macrophages, include acetylation and methylation, and the most frequently modified amino acid is a lysine [178]. Histone modifying enzymes regulate macrophage phenotypes through the addition or removal of acetyl/methyl groups. Acetylation and deacetylation are initiated by histone acetyltransferases (HAT) and histone deacetylases (HDACs), respectively [178]. Histone acetylation is associated with the activation of transcription, whereas histone deacetylation is associated with transcriptional repression. Bromodomain-containing proteins (BRD) and some extraterminal-motif containing proteins (BETs) are also involved in transcriptional regulation by recognizing histone acetylation sites via bromodomain acetyl-binding pocket [175,179]. BETs inhibit or activate the assembly of the transcriptional machinery regulating inflammatory cytokine (IL-1b, IL-6, TNFa, MCP-1) production [175,180]. BRD4 and BRD9 act in the SWI/SNF chromatin remodeling complex in the context of inflammatory stimulation of macrophages [181].

Methylation and demethylation of histones are catalyzed by histone methyltransferases (HMT) and histone demethylases (HDM), respectively. Histone methylation can induce both transcriptional activation and repression, depending on the number and location of the methyl groups [129]. An active transcriptional state is characterized by the presence on the gene promoters or enhancers of activating histone marks such as H3K4me1 and H3K4me3. The repressed state of transcription is associated with the increase in labeling in H3K9me2/me3 and H3K27me3 [182].

Remarkably, histones code acts not only on the promoters, but also on the enhancers that are critical for the differentiation and activation of myeloid precursors and mature macrophages. Single-cell RNAseq demonstrated that various populations of myeloid cells are formed already at the level of bone marrow precursors, that are controlled by a variety of transcription factors (PU.1, Cebp-a, -b and –ε, IRF8, ATF3) [183,184]. The activity of these transcription factors is regulated by histone modifications on the enhancers [183]. Moreover, di- or tri-methylation of histone H3 in lysine-4 and -79 is associated with gene activation, while the methyl group (H3K9me2/3 and H3K27me3) relates to transcriptional repression [183]. Depletion of PU.1 in primary macrophages resulted in the decreased activation of methylation of H3K4 in many enhancers [150]. The importance of gene function regulation using H3K4me2 in enhancers and promoters of IRF8 and CSF1R genes has been established for monocyte progenitors [183]. According to the ChIP-seq data obtained in the projects of the BLUEPRINT consortium, differences between monocytes and macrophages for histones H3K4me3 (promoters), H3K4me1 (enhancers) and H3K27ac (active promoters and enhancers) were revealed [185]. Monocytes gain about 5000 enhancers and lose 3000 enhancers compared with the hematopoietic stem cell (HSC) precursors, while macrophages gain and lose 6000 enhancers when differentiated from monocytes [185]. It was shown that 2547 promoters were changed in histone acetylation status in monocytes compare with macrophages, while a differential pattern of histone acetylation was found in 4036 enhancers [186].

Enzymes that control histone modification, such as HMTs [137,138,139,140,141,143,144,145,146,148,149,150,151,152], HDMs [31,154,155,156,157], HDACs [15,66,121,136,150,152,158,159,160,161,162,163,164,165,166,167,168,169,170,171,172,173], BETs [174,175] are involved in the epigenetic regulation of M1 and M2 macrophage polarization (Table 2, Figure 1). Activation of the TLR-dependent pathway in macrophages and THP1 cells is accompanied by an increase in the expression of the H3K79 inhibitor–disruptor of telomeric silencing-1-like (Dot1l) [187]. SIRT1, a specific type of HDAC, suppresses macrophage activation through TFs such as p65, LXR, and IRF8, and SIRT1 expression is downregulated in LPS-stimulated macrophages [188]. SIRT1 and SIRT2 are rapidly activated during macrophage differentiation, and their inhibition results in the upregulation of many inflammation-related genes. SIRT1 and SIRT2 interact with DNMT3B and bind to the promoters of genes that become hypermethylated during macrophage differentiation that was shown in human macrophages in vitro [161]. IL-4-activated STAT6 acts as a transcriptional repressor in an HDAC3-dependent manner in BMDMs [189].

Most of the data indicates the involvement of histone modification in TAMs in the formation of immunosuppressive M2-like phenotype in tumors (Table 2). For example, activation of extracellular signal–regulated kinases-1/2 (ERK-1/2) results in the inhibition of MyD88 via interleukin 1 receptor-associated kinase 3 (IRAK M) disrupting TLR signaling in TAMs of C57BL/6 mice. Histone phosphorylation of the IL-10 promoter depends on ERK-1/2 and increases IL-10 production, but not IL-12 [190]. BET bromodomain inhibitor, JQ1, blocks the association of bromodomain-containing protein 4 (BRD4) with promoters of arginase and other IL-4-dependent macrophage genes inducing immunosuppression in the TME [191]. When combining JQ1 with a PI3K inhibitor, or using the double PI3K/BRD4 inhibitor SF2523 (previously reported as a strong inhibitor of tumor growth and metastasis in various cancer models), tumor growth was suppressed in syngenic and spontaneous mouse cancer models. This effect was accompanied by the decrease in myeloid suppressor cell infiltration, restoration of the activity of CD8+ T cells, and stimulation of the antitumor immune response [191] (Table 3).

Decoy receptor 3 (DcR3) regulates the expression of HLA-DR in TAMs by affecting the expression of the main regulator of HLA-DR, CIIT-A, through the ERK- and JNK-induced histone deacetylation of CIITA promoters [192]. This is the mechanism responsible for the DcR3-mediated suppression of HLA-DR and polarization of TAMs to M2-like phenotype. The level of DcR3 expression in cancer cells was inversely correlated with HLA-DR expression levels in TAMs and with the overall survival period in patients with pancreatic cancer [192] (Table 3).

The classical (M1) polarization of macrophages is accompanied by a decrease in the expression of lysine-specific histone demethylase 1A (LSD1) (demethylation of H3K4 and H3K9 essential for the myeloid cell differentiation), nuclear REST corepressor 1 (CoREST) and zinc finger protein SNAIL [193]. Treatment with phenelzine (an LSD1 inhibitor) reduced the activity of H3K4 and H3K9 nuclear demethylase that resulted in the activation of the transcription and expression of M1-like markers both in vitro and in vivo in the mouse model of triple negative breast cancer. Additionally, in vivo chemotherapy reduced tumor volume and, in combination with an LSD1 inhibitor, canceled the mesenchymal signature and stimulated an innate M1-like antitumor immune response [100,193] (Table 3).

Histone deacetylases (HDACs) have an ambivalent effect on the regulation of gene expression in TAMs. Pan inhibition of HDAC by suberoylanilide hydroxamic acid (SAHA) reduced NO production in RAW264.7 cells and mouse peritoneal macrophages [194]. SAHA regulates pro-tumor TAM function and induces EMT in prostate cancer cells [194]. The use of this inhibitor together with tritepinoid as anticancer drug led to the decrease in the level of macrophage infiltration into the mammary gland in MMTV-polyoma middle T (PyMT) mice and a subsequent decrease in tumor formation [195]. A similar result was obtained for murine models of lung and pancreatic cancer where inhibition of HDAC had an antitumor effect by acting through the mechanisms of regulation of nitride oxide (NO) production in TAMs [195]. More recently, a class IIa HDAC inhibitor, TMP195, was found to reduce the tumor burden and metastasis by modulating TAM phenotypes to the antitumor, highly phagocytic cells in tumor-bearing MMTV-PyMT mice [203].

Histone modifying enzymes are definitely involved in the cross-talk between cancer cells and TAMs. Thus, Jumonji domain-containing histone demethylases 1A (JMJD1A) regulated by hypoxia and nutrient starvation of cancer cells, stimulates tumor aggressiveness by enhancing the amounts of TAMs and their pro-angiogenic activity [196]. However, whether JMJD1A acts also in TAMs directly still has to be clarified. Despite the accumulated data about the critical role of histone code and histone modifying enzymes in macrophage activation, the role of the histone modifying enzymes in TAM activation in tumor-specific context has to be analyzed for the development of optimal tumor targeting strategy.

### 3.3. microRNA

Small noncoding single-stranded RNAs are evolutionarily conserved and are involved in the multistep processes of transcription, nuclear export and cytoplasmic cleavage [204]. MicroRNAs act primarily as posttranscriptional repressors via the targeting the 3′-untranslated region of mRNA, inducing its degradation or the repression of its translation. More than 60% of all protein-coding genes are directly regulated by microRNAs [114].

Different miRNAs are involved in the regulation of macrophage tumor-supporting and tumor-killing activities. miR-155, miR-181 and miR-451 was found in M1 macrophages and miR-146a, miR-125a and miR-145-5p—in M2 macrophages [117,118], (Table 3, Figure 1). High expression of miR-155, miR-146a, miR-127, miR-125b in M1-polarized macrophages was confirmed in BMDMs isolated from BALB/c mice, the RAW264.7 macrophage cell line and in C57Bl/6 mice [119,205,206] (Table 3, Figure 1). miR-511-3p, miR-223 and let-7c contribute to the polarization of monocyte-derived macrophages into the M2-like phenotype [118,207]. It was demonstrated that increased levels of miR-720 resulted in the inhibition of GATA3 expression, which is important for the polarization of M2 macrophages [29]. Moreover, knockdown of miR-146a promoted polarization of macrophages into M1-like phenotype and decreased polarization to M2-like phenotype [208]. miR-99a inhibits the phenotype and function of M1 macrophages by targeting TNF-α in BMDMs of mice [17]. In P388D1 and RAW264.7 cells miR-511-3p, which was found to be highly expressed in CD206+ macrophages in N202 tumors in mice, regulates the expression of IRF4, thereby supporting expression of genes associated with the M2-like phenotype [201] (Table 3).

In human monocytes stimulated by human larynx epithelioma cancer cell supernatants, and in CD14+ cells obtained from blood of patients with HCC, increased expression of miR-17 and miR-20a resulted in the stimulation of angiogenesis by IL-6-dependent production of hypoxia-induced factor 2α (HIF2a) [200]. Increased expression of miR-511-3p leads to the suppression of the transcriptomic protumoral gene signature detecting by RNAseq in human and mouse CD206+ macrophages, that is associated with the inhibition of tumor growth [202]. In addition, microRNA-19-a-3p inhibits tumor progression by downregulation of human fos-related antigen 1 (FRA-1) gene (acting as a pro-oncogene by supporting the invasion and progression of breast tumors) and the FRA/STAT3 signaling pathway in RAW264.7 cells [202].

Remarkably, in number of epigenetic mechanisms were found to support M2 functions of TAMs that can be explained by the fact that M1 functions are usually activated in the acute phase of inflammation and do not require epigenetic support. The majority of the data are a still coming from the animal tumor models, and a similar role for epigenetic mechanisms in TAMs in human cancers has to be analyzed. The availability of the inhibitors of histone modifying enzymes would be an interesting approach to block M2 polarization of TAMs; however, the specific delivery of such drugs to TAMs, similarly to the delivery of drugs targeting transcription factors, remains to be developed.

## 4. Metabolic Regulation of Macrophage Plasticity

**Figure 2:**
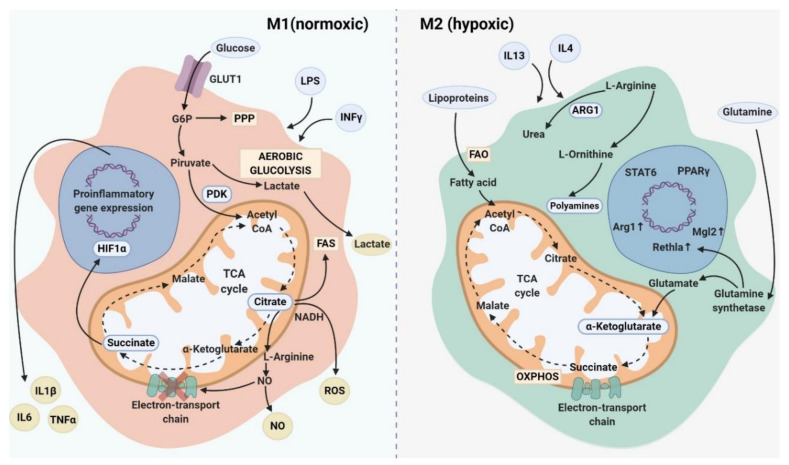
Metabolic characteristics of M1 and M2 macrophages. ARG1—Arginase 1; FAO—fatty acid oxidation; FAS—fatty acid synthesis; G6P—Glucose 6-phosphate; GLUT1—glucose transporter; NADH—Nicotinamide adenine dinucleotide; OXPHOS—oxidative phosphorylation; PDK—Pyruvate dehydrogenase kinase; PPP—pentose phosphate pathway; ROS—reactive oxygen species; TCA—tricarboxylic acid. Figure created in biorender (http://biorender.io).

Numerous studies showed distinct metabolic characteristics for the two main subtypes of macrophages (M1 and M2). Movahedi and colleagues indicated that M1 macrophages are mainly normoxic, while M2 macrophages reside in hypoxic areas of tumor and have a proangiogenic activity in vivo [209]. M1 polarization displays highly glycolytic metabolism through the pentose phosphate pathway (PPP), fatty acid synthesis (FAS) which organizes the plasma membrane for inflammatory signaling, and impaired mitochondrial oxidative phosphorylation (OXPHOS) and tricarboxylic acid (TCA) cycle [210]. It is commonly considered that M1 macrophages are characterized by enhanced antimicrobial activity mediated by the upregulation of reactive oxygen species (ROS), generation of reactive nitrogen intermediates (NO), an increased production of antimicrobial peptides, and pro-inflammatory cytokines, such as IL-1β and TNFα [2,211]. M1 macrophages are able to accumulate both citrate-supported NADPH and prostaglandin E2 (PGE2), and succinate stabilized hypoxia-inducible factor 1α (HIF-1α) [2] (Figure 2). In contrast, traditionally M2 macrophages undergo a metabolic reprogramming toward oxidative metabolism for bioenergetic purposes (OXPHOS), fatty acid oxidation (FAO), decreased glycolysis, decreased metabolism via the PPP and upregulation of arginase 1 (ARG1) which is processed into ornithine to produce polyamines (Figure 2). Such metabolic features are associated with the ability of M2 macrophages to resolve inflammation and to support tissue repair [2,211,212].

However, recent evidences demonstrated that FAO is also essential for inflammasome activation in M1 macrophages, while glycolysis was found to be utilized by M2 macrophages [213]. Below we describe key metabolic pathways of M1 and M2, as well as the examples of mixed metabolism that can be used by macrophages in the complex pathological conditions.

### 4.1. The Key Metabolic Features of M1 Macrophages

It is well accepted that the key feature of inflammatory macrophages is the induction of glycolysis by the up-regulation of the glucose transporter (GLUT1) which mediates glucose uptake [214]. Overexpression of GLUT1, which is a member of GLUT family, in macrophages is associated with increased glycolysis and PPP intermediates that induce ROS production and expression of pro-inflammatory mediators such as TNFα and IL-6 [215]. Overexpression of GLUT1 in murine macrophage cell line RAW 264.1 resulted in elevated secretion of pro-inflammatory mediators, such as G-CSF, IL-6, TNF-α, IL-1ra, increase in ROS production and simultaneously in enhanced glucose metabolism [215]. Moreover, in macrophages, GLUT is controlled by HIF1α which regulates the expression of genes encoding for glycolytic enzymes as well as inflammatory mediators [10]. Thus, the upregulation of GLUT1 promotes glucose uptake that is crucial for the glycolytic activity of M1 macrophages [10,215]. ROS is a prominent factor in the activation of NFkB and p38 MAPK signaling pathways inducing pro-inflammatory gene expression in M1 macrophages [216]. Besides, ROS is involved in the activation of the nucleotide-binding oligomerization domain (NOD)-like receptor containing pyrin domain 3 (NLRP3) inflammasomes [217].

LPS-activated M1 macrophages express 6-phosphofructo-2-kinase B (PFKFB3) and the pyruvate kinase M2 (PKM2) [218]. PKM2 was found to activate the LPS-induced pro-inflammatory phenotype of M1 macrophages in murine model via the production of HIF-1α, IL-1β and other HIF-1α-dependent genes as well as to promote inflammasome activation by modulating eukaryotic translation initiation factor 2 alpha kinase 2 (EIF2AK2) phosphorylation in macrophages [218,219]. Pyruvate dehydrogenase kinase 1 (PDK1) was demonstrated as a critical component of glucose metabolism, which was involved in LPS-induced macrophages activation [220]. Knockdown of PDK1 in murine BMDMs suppressed M1 by attenuating glycolytic flux, the expression of pro.inflammatory cytokines (TNF-α and IL-6) and consequently aerobic glycolysis, but enhanced M2 activation by mitochondrial respiration [220]. Moreover, combined deletion of two forms of pyruvate dehydrogenase kinase PDK2 and PDK4 in myeloid cells prevents M1 polarization and correlates with the improved mitochondrial respiration in mouse models [221]. Similarly, PDK1 was identified as a HIF-1α target gene, and HIF-1α-PDK1 axis induced active glycolysis with up-regulation of glycolytic genes, such as GLUT1, phosphoglycerate kinase 1 (PGK1) or lactate dehydrogenase A (LDHA) [222].

However, there are recent evidences about the crucial need for glycolysis in M2-like macrophages both for the activation of M2-specific gene expression and for the tumor support [223,224]. Analysis of different components of Akt signaling revealed that Akt mediates enhanced glucose consumption in murine IL-4-stimulated BMDMs [225]. Depletion experiments showed that IL-4 treatment enhanced global acetylation of H3 and H4 histones at promoters of M2 genes (ARG1, Retnla, MGL2) in an Akt-mTORC1-dependent manner. Moreover, Akt controls the production of Ac-CoA, the metabolic substrate for histone acetylation. Inhibition of histone acetylase p300 as well as knockdown of Raptor, a main subunit of the mTORC1 complex, reduced induction of Akt-dependent M2 genes [223]. Increased aerobic glycolysis was also found in murine BMDMs synergistically stimulated with M-CSF and IL-4 [226]. Glycolysis and mitochondrial pyruvate import were essential for M2 activation, possibly because they were used to fuel FAS for increased FAO and OXPHOS. mTORC2-mediated phosphorylation of Akt was critical for M2 activation. Deletion of Rictor, a subunit of mTORC2 complex, diminished the expression of a number of M2-specific genes (CD301, RELMα, ARG1, Chil3 (Ym1), IL-10, LIPA, CD36, FABP4, PPARG, and PPARGC1B) and glucose uptake in IL-4-stimulated macrophages. Besides, Rictor-deficient macrophages showed inhibition of activity of transcription factor IRF4, indicating the role of mTORC2 in the expression of IRF4 in IL-4-stimulated macrophages. In an in vivo mouse model of melanoma, loss of the mTORC2 in TAMs diminished M2 activation and suppressed tumor growth [224]. Interestingly, in vitro knockdown experiments revealed that STAT6 and Akt-mTORC signaling may operate in parallel and independently in response of BMDMs to IL-4 [223,224]. Despite that it is well-known that Akt-mTORC signaling is involved in the regulation of glucose consumption and glycolysis, there is limited evidence about regulating glucose metabolism via STAT6 activation [225,226]. Further investigations of the interaction of these two significant pathways in the regulation of glucose metabolism are urgently needed.

The metabolic value of pentose phosphate pathway (PPP) in M1 polarization includes conversion of glycolytic intermediates to precursors of nucleotides and amino acids. The PPP generates NADPH required for the inducible nitric oxide synthase (iNOS) to catabolize arginine into nitric oxide (NO) and l-citrulline as well as for the generation of ROS [227,228]. Suppression of PPP in macrophages attenuates oxidative stress responses and LPS-induced inflammatory cytokines that were shown in a hyper cholesterolemic mouse model [229].

A truncated TCA cycle was considered as a metabolic feature of M1 macrophages leading to the accumulation of citrate and succinate [230,231,232]. Citrate can be involved in fatty-acid synthesis, which is essential for membrane biogenesis [230], and in the generation of inflammatory effector molecules such as NO and prostaglandin that negatively modulate mitochondrial activity by disrupting electron transport chain [231,233]. Pyruvate dehydrogenase (PDH) activity is needed to synthesize citrate from glucose-derived pyruvate, while citrate is used for lipogenesis and for the production of the pro-inflammatory mediators such as NO [210]. Succinate is associated with the pro-inflammatory function of M1 macrophages [210]. LPS-induced succinate in macrophages enhanced IL-1β production by stabilizing HIF-1α [232]. Succinate may indirectly stabilize HIF-1α via the induction of ROS [210].

Moreover, hyperglycemia was found to induce production of pro-inflammatory cytokines and S100 proteins in human primary macrophages [234,235,236]. One of major pro-inflammatory cytokines is IL-1beta that has a complex role in tumors and promotes tumorigenesis, tumor invasiveness and immunosuppression [237,238]. S100A9 and S100A12 that are induced by high glucose in primary human macrophages have multiple cellular targets and link inflammatory processes in cancer [239]. We have recently demonstrated that hyperglycemia induces activating histone code on the promoters of these genes in primary human macrophages, that shows that there is a link between glycolytic metabolism and the epigenetic level of regulation in macrophages [236]. However, it remains to be understood how these processes interact in TAMs.

### 4.2. The Key Metabolic Features of M2 Macrophages

A key metabolic signature of alternatively activated macrophages is the consumption of fatty acids and the increase in the mitochondrial oxidative phosphorylation (OXPHOS) [210]. Using BMDMs from CD36–/– mice it was shown that the uptake of low-density lipoproteins (LDL and VLDL) is mediated by the scavenger receptor CD36 leading to their subsequent liposomal lipolysis activating OXPHOS and FAO in M2 macrophages. Furthermore, elevated CD36 expression is substantial for the up-regulation of gene expression defining for IL-4-induced macrophages (CD206, CD301, PD-L2 and RELMαin) [240]. Surprisingly, FAO was detected as the key metabolic process involved in inflammasome activation, a key signaling event in pro-inflammatory macrophages. Inhibition of FAO by etomoxir treatment suppressed NLRP3 and consequent secretion of IL-1b and IL-18 in human and mouse macrophages [241]. FAO was shown to be required for palmitate-induced NLRP3 inflammasome activation, which involves mitochondrial ROS [242]. Additionally, in vivo delivery of CpG oligodeoxynucleotide, a Toll-like receptor 9 agonist, to tumor-bearing mice with pancreatic ductal adenocarcinoma (PDAC) cells resulted in the suppression of tumor growth in pancreatic cancer models enhancing the anti-tumor activity of F4/80+ TAMs through the induction of phagocytosis of tumor cells [243]. The anti-tumor activity of TAMs is implemented by the upregulation of FAO that is a key feature of M2 macrophage metabolism, however increased pro-inflammatory cytokines (TNF, IFNγ and CCL2) in the serum of mice were also detected. FAO inhibition by etomoxir did not alter the abundance of F4/80+ macrophages in the tumor microenvironment, however, it was associated with decreased engulfment of PDAC cells by F4/80+ macrophages [243].

These numerous studies demonstrated the regulation of M2 polarization of macrophages through the impact on the key metabolic pathways. Interestingly, simultaneous stimulation with LPS and IFNγ blunted mitochondrial oxidative respiration in macrophages which cannot be restored by subsequent IL-4 stimulation that was demonstrated in mouse BMDM and human monocyte-derived macrophages [233]. The main metabolic effect was accompanied by NO which impeded M1→M2 repolarization by blunting mitochondrial respiration and preventing plasticity in M1 macrophages. Inhibition of NO improved mitochondrial function and promoted IL-4-induced repolarization of M1 into M2 [233].

Thus, macrophage metabolism is not strictly limited to the glycolysis in M1 and FAO in M2 phenotypes, and examples of mixed metabolism in macrophages were also identified [210]. However, most studies are based only on in vitro data, and analysis of TAM metabolism in mouse tumor models and in patient’ material is needed to understand the complex metabolic response of macrophages to the stimuli of microenvironment in various types of cancer, and the role of TAM metabolism in their pro- and anti-tumor activities.

### 4.3. Metabolic Interactions of TAMs and Cancer Cells

In the tumor microenvironment cancer cells adapt their cellular metabolism to the hypoxic conditions to maintain a high proliferation rate and invasive activity. Tumor is highly limited in the energy suppliers, and cancer cells and other cells of TME compete for the oxygen and nutrients [2]. The altered metabolism of cancer cells is called the Warburg effect and is characterized by an increase in glycolysis even under aerobic conditions [227]. Cancer cells preferentially convert pyruvate into lactate. TAMs can respond to the products of altered cancer cell metabolism by changing their functional program to support tumor progression and metastasis [214].

#### 4.3.1. Role of Lactate and Glycolysis

Growing evidence indicates that extracellular accumulation of lactate produced by cancer cells stimulates expression of pro-angiogenic and tumor-promoting factors in TAMs, and consequently induces TAM-mediated immunosuppression [244,245]. The importance of lactate in the activation of tumor-promoting activity of TAMs was demonstrated in co-culture system of human monocytic cell line THP-1 with MDA-MB-231 and MCF-7 human breast cancer cells [67]. Lactate programmed TAM-like phenotype of THP-1 cells (upregulation of CD206 and CD163 expression and elevated production of TGF-b1, IL-10, VEGF) and stimulated the expression and secretion of CCL5. CCL5, in turn, induced an invasive phenotype of breast cancer cells by enhancing migration, EMT and aerobic glycolysis [244]. The pro-metastatic phenotype of macrophages was also shown in a model system of TAMs differentiated from human monocytes in the presence of conditioned medium of the pancreatic ductal adenocarcinoma cells (PDAC) [245]. TAMs promoted vascular network formation and supported EMT and extravasation of cancer cells. PDAC conditioned medium stimulated glycolysis in macrophages by up-regulation of a number of glycolytic genes, including hexokinase (HK2), glucose-6-phosphate isomerase (GPI), aldolase A (ALDOA), triosephosphate isomerase 1 (TPI1) and phosphoglycerate kinase 1 (PGK1). Inhibition of glycolysis in TAMs using inhibitor of HK2, 2-deoxiglucose (2DG), significantly suppressed pro-metastatic phenotype of TAMs [245]. Analysis of TAMs from MMTV-PyMT mice and BMDMs stimulated by tumor extract from MMTV-PyMT mice revealed the significant increase in HK2, enolase 1 (ENO1), and 6-phosphofructokinase (PFKL), a key mediators of aerobic glycolysis [246] (Table 4).

There are also evidences of TAM-dependent metabolic re-programming of tumor cells to aerobic glycolysis. For example, in human breast cancer tissues the positive correlation between CD68+ TAM infiltration and glycolytic enzyme expression GLUT1, GLUT3 and HK2 in cancer cells was demonstrated by immunostaining [261]. In the same study, MDA-MB-231, MDA-MB-468, MCF-7 and BT474 breast cancer cells co-cultured with TAMs polarized by conditioned medium from breast cancer cells showed enhanced aerobic glycolysis by the increase in extracellular acidification rates (ECARs), glucose consumption and lactate production [261]. Besides, breast cancer cells co-cultured with TAMs showed high expression of glycolytic enzymes, including GLUT3, HK2, PKM2 (pyruvate kinase isozyme M2) and LDHA. In this case, activated aerobic glycolysis in breast cancer cells is mediated by stabilizing HIF-1α protein [261]. TAM-enhanced aerobic glycolysis in cancer cells was also shown in lung cancer [262]. A strong correlation between CD68+ macrophages and the expression of GLUT1 and HK2 in cancer cells was found in patients with non-small-cell lung carcinoma (NSCLC) [262]. In the same study, Lewis lung cancer (LLC)-cells co-cultured with BMDMs showed active glycolysis and increased lactate production. TAM-derived TNFα facilitates glycolysis and inhibits mitochondrial biogenesis in LLC cells [262]. Moreover, TAMs can compete for oxygen with cancer cells contributing to tumor hypoxia. In LLC mouse model, TAMs isolated out of tumor expressed significantly increased levels of hypoxic factors including VEGFR, Slc2a1, PDK1, and C-X-C motif chemokine receptor-4 (CXCR4), M1-polarized marker (NOS-2) as well as M2-polarized marker (ARG1), and immunosuppressive cytokines such as TNFa and IL-10. Depletion of TAMs switched the tumor metabolism from aerobic glycolysis to OXPHOS, significantly decreased expression of glycolytic gene, reduced the amount of lactate, and decreased GLUT1 protein expression [262] (Table 4).

AKT1/mTOR pathway is important for activation of glycolysis in TAMs [223]. Mechanistic target of rapamycin (mTOR) complex 1 (mTORC1) inhibitor REDD1 (regulated in development and in DNA damage response 1) was up-regulated in hypoxic TAMs of a murine model of LLC [249]. Inhibition of mTORC1 by REDD1 resulted in the shift of the macrophage phenotype towards the immunosuppressive and pro-angiogenic phenotype that was due to the inhibition of glucose uptake and glycolysis and enhancing glucose availability for endothelial cells. REDD1 deletion in TAMs from murine LLC tumor promotes tumor vessel normalization and inhibits metastasis, providing evidence about the link between TAM metabolism in hypoxia and tumor vessel morphogenesis [249].

In an in vitro model of TAMs where human blood monocytes were stimulated with the conditioned medium of human melanoma cells (MV3), TAMs expressed M2 specific markers (CD206 and CD163), however they were metabolically distinct from typical M2 and had metabolic features of M1-like macrophages. TAMs polarization resulted in the increased GLUT1 and HK2 expression, increased glycolysis, and high amounts of lactate by Akt–mTOR-dependent pathway that was comparable with M1 macrophages. In parallel, TAMs were characterized by the supporting OXPHOS, presenting a high basal and maximal oxygen consumption rate (OCR), while showing low rates of FAO [247] (Table 4). This study showed that macrophages can produce lactate in response to soluble factors from condition medium of tumor cells, however the role of TAM-derived lactate in tumor progression remains to be identified.

Immunohistochemical analysis (IHC) of tumors of patients with thyroid cancer (TC) also validated the increase in glycolytic enzymes and lactate receptor (GBR18, PFKFB3, PKM2) in TAMs [248]. Stimulation of human macrophages with TC-conditioned medium or co-cultivation of macrophage with TC cells induced increased glycolysis in human macrophages by elevation of ECAR in an mTOR-dependent manner. RNA-sequencing confirmed on the transcriptional level enhanced expression of genes regulating glycolysis in TAMs [248].

A combination of lactate and hypoxia in TME results in the induction of ARG1 expression and increased secretion of VEGF-A by ischemic macrophages [250]. In an MMTV-PyMT mouse model of breast tumor, TAM-derived VEGF were required for the response of endothelial cells for vascular morphogenesis [250]. Interestingly, in breast cancer tissue TAMs expressing CD206 are located in well-nourished perivascular regions, whereas macrophages produced high levels of ARG1 located within hypoxic regions, far from the vasculature [250,251]. Upregulation of ARG1 in TAMs results in the production of polyamines critical for the stimulation of cancer cell proliferation [251].

Thus, not only tumor cells-derived lactate stimulates tumor-promoting function of TAMs, but in turn, cancer cell-activated macrophages activate aerobic glycolysis in cancer cells leading to their survival, proliferation, and long-term maintenance. Such a metabolic feedback loop provides beneficial conditions for tumor progression.

#### 4.3.2. Role of Hypoxia

In different in vitro models hypoxia stimulated expression of HIF-inducible pro-angiogenic genes, such as VEGF, basic fibroblast growth factor (βFGF) and CXCL8, as well as glycolytic enzymes in TAMs [263,264]. As a rule, macrophages infiltrate hypoxic regions in tumors in association with increased expression of pro-migratory factors CCL2, CCL5, CSF1 [265]. It was shown that melanoma cancer cells in vitro released damage-associated molecular pattern High-Mobility Group Box 1 protein (HMGB1) in response to hypoxia [252]. HMGB1 is significantly increased in metastatic melanoma in patients, and drives the accumulation of M2-macrophages with elevated expression of YM1, FIZZ1, IL-10 in murine model of melanoma. However, the depletion of HMGB1 with shRNA in mice with B16 melanoma cells-derived tumor significantly reduced tumor growth and the amount of TAMs [252]. The significant influence of hypoxia was shown in macrophages differentiated in vitro from human peripheral blood or BMDMs isolated from mice bearing deletions in the HIF-1α or HIF-2α genes [266]. Under the hypoxia condition, primary human and murine macrophages displayed the upregulation of the cell surface receptors, CXCR4 and GLUT1, and tumor-promoting cytokines VEGFA, IL-1β and IL-8, adrenomedullin, CXCR4 and angiopoietin-2, indicating the importance of both HIFs 1 and 2 in response of macrophages to hypoxia [266].

Hypoxia-inducible factors (HIFs) play a key role in the regulation of cellular responses to hypoxia. Notably, up-regulation of HIF1α promotes immunosuppressive activity of TAMs and differentiation of MDSCs to TAMs [267]. LPS was found to activate HIF-1α in murine AHA-1 macrophage cells under hypoxic conditions in vitro. LPS induced transcriptional activity, but not protein expression and DNA binding activities of HIF-1α in macrophages by a ROS-dependent pathway [268]. It was shown that hypoxia influences mitochondria electron transport chain (ETC) and drives ROS increase by acting on complexes I, II, and III of the ETC [269]. Although ROS is a key metabolic marker of M1 polarization, it was shown to play a crucial role in the differentiation of monocyte to M2 macrophages in response to M-CSF and IL-4 in vitro [270]. Inhibition of ROS generation by antioxidant butylated hydroxyanisole (BHA) specifically affects the polarization of macrophages to M2, and dramatically inhibits the expression of the M2 cytokines IL-10, CCL17, CCL18 and CCL24, but not M1 cytokines. Additionally, ROS inhibitor BHA significantly reduced the accumulation of F4/80+cells and tumor burden as well as numbers of metastatic foci in K-RAS-induced lung cancer and MMTV-PyMT-induced breast cancer in vivo [270]. ROS production is regulated by NADPH oxidases. NADPH oxidase 4 (NOX4)-overexpressed lung cancer cell lines A549 and Calu-1, induced the recruitment of murine M2-like TAMs via the ROS/PI3K signaling-dependent pathway [253]. ROS produced by cancer cells stimulates various cytokine production, including CCL7, IL-8, CSF-1 and VEGF-C, that all contribute to enhanced NSCLC cell growth. IHC analysis of clinical specimens confirmed the positive correlation of NOX4 and CD68 or CD206 [253]. ROS accumulation in BMDMs that was reached by ROS inducer, glutathione synthesis inhibitor buthionine sulphoximine (BSO), results in increased expression of programmed death ligand-1 (PD-L1) and production of IL-10, IL-17, IL-4, IL-1b, insulin-like growth factor-binding protein 3 (IGFBP-3), and chemokine (C-X-C motif) ligand 1 (CXCL1) that are associated with an immune-suppressive phenotype of macrophages [271].

In another study, hypoxia promoted THP-1 cells polarization to M2 phenotype in HIF-1α-independent way, by decreasing IL-1β expression and increasing VEGF and CD206 expression [272]. In patients with glioma, IHC analysis revealed the positive correlation between HIF-1α expression, periostin (POSTN) expression, and the infiltration of TAMs (CD11b+) and M2 type TAMs (CD206+) in tumor sections. The density of TAMs increased in higher grade gliomas and in hypoxic HIF-1α-positive regions. In vitro supernatants from hypoxia-treated U87 or U251 glioma cell induced strong chemotactic effect toward THP-1 cells, upregulation of M2 marker expression (IL-10 and CCL-22) and downregulation of M1 markers (IL-6 and TNF-α), indicating the activation of M2-like phenotype under hypoxic condition. Hypoxia-inducible expression of POSTN, tumor-promoting factor and chemoattractant for macrophages, in U87 and U251 cells was increased by TGF-α via the RTK/PI3K pathway in vitro [254]. Conversely, in the model of lung adenocarcinoma, hypoxia induced the metabolic shift in TAMs from glycolysis toward TCA cycle and OXPHOS activation [255]. Thus, exosomes derived from hypoxic B16-F0, A375, A431, and A549 lung adenocarcinoma cells were highly enriched with CSF-1, CCL2, FTH, FTL, and TGFβ that induced macrophage recruitment and promoted M2 polarization. In vivo, exosome-treated BMDMs showed a shift of cell population to F4/80+CD206+ population, increased B16-F0 tumor cell proliferation and viability. ATP-linked mitochondrial OCR assay demonstrated that M2-like macrophages, polarized by hypoxic exosomes, exhibited enhanced OXPHOS activity, inhibiting AKT and mTOR and increasing expression levels of mTOR negative regulator REDD1 [255]. These numerous studies indicate that hypoxia promotes the tumor-supporting function of TAMs, which is associated with a strong induction of immunosuppressive and proangiogenic phenotype.

#### 4.3.3. Role of Fatty Acid Oxidation (FAO)

The importance of FAO for TAMs has been recently shown in a murine colon carcinoma model and in human colon cancer where unsaturated fatty acids (oleate) induced polarization of immunosuppressive TAMs by supporting mitochondrial respiration [257]. The up-regulation of M2 specific markers (CD206, IL-6, VEGFα, MMP9, ARG1) was observed upon oleate treatment dependent on lipid droplets (LD), that play an essential role in the catabolism of free fatty acids for mitochondrial respiration. The formation of LDs in TAMs was found in tumor tissue of patients with colon cancer [257]. However, inhibition of DGAT, an enzyme responsible for the formation of lipid droplets in myeloid cells, prevented oleate-induced immunosuppressive M2 phenotype in murine BMDMs and human monocyte-derived macrophages. Besides, mTOR inhibition in myeloid cells eliminated specific lipid droplet-dependent mitochondrial respiration in M2-like macrophages [257]. In contrast, the LD formation in TAMs from a mouse mammary adenocarcinoma model was associated with significantly inhibited tumor growth. LDs were formed particularly in M1-like (MHCII+CD11c+) TAM population in E0771 breast cancer-bearing mice. This subset of macrophages demonstrated up-regulation of epithelial fatty acid binding proteins (E-FABP), a lipid chaperon. Furthermore, the expression of E-FABP in human breast tumors is reduced in macrophages of invasive tumors as compared to normal stroma, and decreased TAMs in parallel with the disease progression [258]. IFNγ induces LD accumulation in MafB/c-Maf double deficient (Maf-DKO) macrophages that depends on exogenous lipids, while de novo synthesis of fatty acids from glucose plays a minor role in this process [273] (Table 4).

Other pathways are also involved in metabolic changes in TAMs. Thus, TAMs isolated from human renal cell carcinoma produce pro-inflammatory chemokine CCL2 and immunosuppressive cytokine IL-10 that is dependent on the increased metabolism of 15-lipoxygenase-2 (15-LOX2) LOX-dependent arachidonic acid [259]. TAMs isolated from tumor-bearing mice (B16 melanoma and ID8 ovarian carcinoma) induced itaconate accumulation which is catalyzed by the enzyme encoded by immunoresponsive gene 1 (IRG1) [260]. Itaconic acid stimulates OXPHOS and ROS production in TAMs. Interestingly, IRG1 protein expression was found in TAMs from tumor-bearing mice, but was not detected in B16 or ID8 tumor lysates, and Irg1 shRNA treatment significantly reduced tumor burden in both tumor models. These results indicate once again that tumors profoundly alter the metabolism of TAMs, to potentiate tumor growth [260]. Other authors reported glutamine-synthetase (GS) as mediator of the proangiogenic, immunosuppressive, and pro-metastatic M2-like macrophages. It was reported that glutamine-synthetase (GS) controlled mTOR signaling and activated IL10-stimulated M2 macrophages with pro-tumor properties [256]. Moreover, deletion of GS in macrophages promotes vascular normalization, accumulation of cytotoxic T cells, and metastasis inhibition and skews TAMs toward the M1-like phenotype in mice implanted with Lewis lung carcinoma (LLC) cells. Deletion of GS in macrophages leads to the reduced expression of M2-specific markers (ARG1, CD206, CCL17, and CCL22) and upregulation of M1 marker MHC class II [256]. GS-targeted human monocyte-derived macrophages display reduced glutamine and enhanced succinate accumulation, increasing glucose flux through glycolysis, partly through the stabilization of HIF-1α [256]. The elevated expression of GS was also revealed in TAMs isolated from glioblastoma resections and TAMs co-cultured with glioblastoma cells [274].

Thus, the available data indicate that tumors can program the metabolism of intratumoral macrophages to potentiate tumor growth. Although the molecular profile of TAMs is very close to M2-prototype, in TME they obtain mixed metabolism with pronounced glycolysis, a metabolic feature of M1 macrophages. Among the number of metabolites in TME, the essential tumor-promoting role of TAMs in different cancer models was assigned to lactate released by cancer cells. Lactate increases the ability of TAMs to induce angiogenesis, tumor growth and immunosuppression. The importance of FAO, a metabolic feature of M2 macrophages, has been also demonstrated in TAMs. However, there are some contradictory results concerning the lipid droplets involved in fatty acid metabolism. The majority of studies were performed using in vivo or in vitro models, and almost no results can be found for patients. Analysis of TAM metabolism in human tumors is required in order to find therapeutic targets to stimulate the anti-tumor activity of TAMs.

## 5. Conclusions and Perspectives

Each tumor is a complex organ with individual dynamics of growth, metabolism, immune status, vascularization and spread within the organism. Macrophages are key innate immune cells in the TME and at metastatic sites that have the intrinsic capacity to block cancer progression, but in the majority of tumors they are reprogrammed by cancer cells to support tumor growth and spread. Programming of macrophage functional phenotypes is controlled on the transcriptional, epigenetic, and also on the metabolic levels. Close interplay of transcriptional factors and epigenetic enzymes is responsible for the activation of pro- or anti-tumor programs, and is utilized by cancer cells to give instructions to macrophages to support tumor progression. The progress in our understanding of essential elements and mechanisms that control interaction between transitional factors and epigenetic mechanism in complex TME resulted in the identification of a promising target for therapy. For example, inhibition of some TFs, such as STAT3 or STAT6, c-Maf, c-Myc, in macrophages can significantly attenuate tumor growth and metastasis of tumors [21,77,78,79,106].

Metabolism of macrophages attracted more recently strong attention of the research community mostly due to the role of macrophages in development of diabetes and its complications. However, cancer cells can control macrophage activation also by modulation of their metabolic pathways. Despite that TAMs are considered to have an M2 phenotype; in TME they can have mixed metabolism with pronounced glycolysis, a metabolic feature of M1 macrophages, and less pronounced FAO. Metabolic re-writing is an attractive idea for therapeutic inhibition of tumor-promoting activity of TAMs but needs a deep understanding of which types of metabolism (glycolytic or FAO) are beneficial for the tumor and which for the patient.

There are several immunomodulatory approaches based on the targeting of macrophage metabolism. A clinical study based on the administration of oleic acid combined with Vitamin D-binding Gc-globulin-derived macrophage activating factor (GcMAF) in patients with advanced cancer (including colorectal cancer, breast cancer, melanoma, thyroid cancer, renal carcinoma) was performed [275]. Administration of the OA-GcMAF complex resulted in a significant reduction in tumor size, demonstrating greater anticancer effects and immunotherapeutic activity than GcMAF alone. One of the possible mechanisms of this effect is releasing of NO responsible for the anti-cancer properties of activated macrophages [275]. Another vitamin D binding protein-macrophage activating factor (DBP-maf) was demonstrated to inhibit the growth of hepatocellular carcinoma in tumor-bearing severe combined immunodeficiency (SCID) mice [276]. In vitro DBP-maf inhibited the proliferation of endothelial cells and activated phagocytosis by macrophages [276]. Targeting glutamine metabolism using glutamine antagonist JHU083 demonstrated the inhibition of metastasis and enhanced anti-tumor immunity in 4T1 (breast cancer) tumor-bearing mice resulting in the improvement of the efficacy of anti-PD1 and anti-CTLA4 therapy [277]. Glutamine antagonist JHU083 induced the repolarization of MDSCs to inflammatory macrophages and enhanced immunogenic tumor cell death and antigen presentation of TAMs [277].

In conclusion, understanding the complexity of the mechanism of the interaction between transcriptional, epigenetic and metabolic programming of macrophages is the next challenge that will allow identifying pharmacological targets for immunomodulatory therapy in specific tumor types. However, the development of delivery systems for specific targeting for pro-tumoral TAMs in different types of cancer is the next task for biotechnology.

## Figures and Tables

**Table 1:** The role of transcription factors in macrophage polarization.

Transcription Factor	Induced by	Role in M1/M2	Role in TAM Activation	References
c-Maf	IL-10	In M2: activation of M2-related genes IL-12, IL-1b, IL-6, ARG1, IL-10, VEGF, TGFb, IRF4, and CCR2 in mouse BM	Activation of CD115, CD301 and inhibition of IFN-γ in TAMs	[19]
c-Myc	IL-4	In M2: activation of genes: SCARB1, ALOX15, CD206 and TFs (STAT6 and PPARγ) in human macrophages stimulated by PANC-1-conditioned medium; activation of Wnt/β-catenin signaling in mature murine BMDMs cultured in conditioned medium of Hepa1-6 cells	Activation of tumor-supporting factors (VEGF, MMP9, HIF1α) in TAMs in a mouse model of melanoma	[20,21,22,23]
IRF1	IFNγ/LPS	In M1: activation of genes: IL-12, IL-6, IL-23, CD86;’ In M2: suppression CD206 in human macrophage cell line U937	TAM-mediated inhibition of proliferation and invasion of HepG2 and SMMC-7721 cells lines	[24]
IRF3	M-CSF/LPS	In M1: inhibition of genes: IL-1α, IL-1β, TNFα, IL-6, IL-8, and CXCL1; In M2: stimulation of genes: IL-1RN, IL-10, and IFN-β via PI3K/Akt signaling pathway in human fetal microglia; suppression of VEGF and MMP2 in IRF3- and IRF7-transduced macrophages	Activation of IL-10, TGFβ and IFN-inducible chemokines (CCL5, CXCL9, CXCL10, and CXCL16) in TAMs isolated from murine fibrosarcoma; Polarization of TAMs in M1 type via TLR3- and TLR4-IRF3 signaling	[25,26,27,28,29,30]
IRF4	IL-4/IL-6	In M2: activation of genes ARG1, FIZZ1, Ym1, and CD206 in mouse BMDMs	Activation of CD163, FN1 and IRF4 in TAMs associated with reduced survival in samples from renal cell carcinoma patients	[31,32]
IRF5	LPS/IFNγ	In M1: activation of gene expression (IL-12p40, IL-12p35 and IL-23p19) In M2: inhibition of anti-inflammatory genes	Reduced tumor development in advanced-stage ovarian cancer, metastatic melanoma, and glioblastoma	[33,34]
IRF7	IFNγ/IL-10	In M2: suppression of VEGF and MMP2 in IRF3- and IRF7-transduced macrophages; In M1: supression of LPS-induced IL-10 production in human MDMs	Decreased expression of VEGF and MMP2 and cytotoxic activity regarding breast cancer and colorectal cancer cell lines; Activation of Wnt/β-catenin signaling	[30,35]
IRF8	IFNγ	In M1: activation genes IL-1b, IL-6, iNOS, and TNF-α in macrophages In M2: no impact on ARG1,MRC-1, IL-10 in mouse macrophages	Decreased metastasis and increased survival in human breast, lung cancers and melanoma. Associated with prolonged DFS in RCC patient	[18,36,37]
KLF4	IL-4	In M1:suppression of LPS-induced expression of NF-κB target genes (iNOS, IL-1β, TNFα and IL-6) in mouse macrophages In M2: activation of ARG1 and FIZZ1 in murine peritoneal macrophages	Suppression of M2 markers (ARG1, CD206, IL-10, TGF-β1, and Chil3) in TAMs and reduction of hepatocellular carcinoma growing in murine myeloid cells; Cooperation with STAT6 promotes MFHAS1-induced M2 polarization of TAMs and tumor progression in murine model of colorectal cancer	[38,39]
KLF6	LPS/IFNγ	In M1: activation of IL-1α, IL-1β, TNF-α, MCP-1, COX2 и MIP-1; cooperates with NF-kB;Suppression of iNOS, IL-1β, TNFα and IL-6 in murine peritoneal macrophages; In M2: supression of ARG1, PDCD1Lg2, MRC1, Chi3l in RAW264.7	Role unknown	[40,41]
MafB	IL-10 or IL-4/IL-13	In M2: up-regulation of MMP9 and IL-7R genes via the IL-10/STAT3 signaling pathway	Activation of IL-10, ARG1 and TNF-a in TAMs in LCC tumors of MafB-GFP knock-in heterozygous mice	[42,43,44]
NFAT5	IFNγ/LPS	In M1: activation of iNOS, IL-12p40 and IL-6 in mouse BMDMs	Activation of infiltration of CD8+ T cells, reduced tumor growth in LLC and ID8 murine models	[45]
NF-kB	LPS/IFNγ/IL-17	In M1: activation of M1-specific genes	Activation of IL-10, TNF-α, and ARG1 and inhibition of NOS2, IL-12, and MHC II, that was mediated by the IL-1R and MyD8 in TAMs increases tumor invasiveness and tumor growth in ovarian cancer in vivo; Differentiation in M2-like phenotype (increasing TGF-β, VEGF and IL-10)	[46,47]
PU.1	IL-4	In M1: inhibition of COX-2, iNOS, TLR4, IL-6, MCP-1, IL-1β, TNF-α, and neutrophilic chemokine keratinocyte-derived chemokine; In M2: activation of IL-4/STAT6 signaling pathway in murine BMDMs; Activation of Ym-1 and Fizz-1 in murine macrophage; Activation CCL22 in murine BMDMs	In patients with breast cancer high expression of PU.1 is associated with shorter survival	[48,49,50,51,52]
SNAIL	TGF-β	Through the PI3K/AKT and Smad2/3 signaling pathways	Overexpression of SNAI in human head and neck cancer cells promotes M2 polarization of TAMs by delivering MiR-21-abundant exosomes	[53]
STAT1	LPS/IFNγ	In M1: activation of IFNγ and TLR signaling pathways	Increasing of CD68+pSTAT1+ cells in patients with cervical cancer is associated with a longer DFS and OS; Induction of immunosuppressive activity of TAMs in tumor-bearing BALB/c mice	[54,55,56]
STAT3	IL-4/TGFβ1/IL-6/IL-10	In M2: Wnt3a exacerbates STAT3-mediated M2 polarization in RAW264.7 cells and BMDMs; activation of IL-6/STAT3 signaling pathway in co-culture of HCC and macrophage	Increasing angiogenesis via induction of angiogenic factors (VEGF and bFGF) in tumor-associated MDSC; Cooperation with STAT6 enhances tumor invasion in vivo via increasing cathepsin expression in TAMs	[57,58,59,60]
STAT6	IL-4/IL-13/IL-6/IL-13	In M2: stimulation of IL-4 and/or IL-13, mediators of Th2 immune responses	Cooperation with STAT3 enhances tumor invasion in vivo via increasing cathepsin expression in TAMs; Inhibition of TRIM24 protein modulates polarization of macrophages toward the TAMs in murine model of melanoma; Cooperation with KLF4 promotes MFHAS1-induced M2 polarization of TAMs and tumor progression in murine model of colorectal cancer; Activation of JAK2–STAT6-GATA3 signaling in TAMs facilitates metastatic colonization in murine mammary carcinoma	[61]
TFEB	LPS	In M1: activation of iNOS, TNF-α; In M2: inhibition of ARG1 and YM-1 in mouse peritoneal macrophages	Suppression of TAM polarization in M2 type and inhibition breast tumor growth in mice; Inhibition of angiogenesis, tumor growth and immunosuppresion through downregulation of STAT3 in co-culture of breast cancer cell line and macrophages	[62]

Notes: BMDMs—bone marrow-derived macrophages; DFS—disease-free survival; HCC—hepatocellular carcinoma; LLC—Lewis lung carcinoma; LPS—lipopolysaccharide; MDMs—monocyte-derived macrophages; OS—overall survival; RCC—renal cell carcinoma.

**Table 2:** Epigenetic effectors involved in macrophage polarization to M1 or M2 direction.

Epigenetic Enzyme Family	Enzyme	Regulation of Macrophage Function	References
DNMT (DNA methyltransferases)	↑DNMT1	Increase in TNFa and IL-6 via the SOCS1 silencing	[133]
	↑DNMT3b	Increase in TNFa production and decrease in ARG1, CD206, MGL1 levels by PPARɣ promoter methylation	[132]
TET (ten-eleven translocation proteins)	↓TET2	Upregulation of inflammatory mediators, including IL-6, during the response to LPS	[136]
HMT (histone methyltransferases)	↑PRMT1	In M1: over-expression of PRMT1 repressed MHCII promoter activity via CIITA methylation;In M2: regulation of PPARγ gene expression through histone H4R3me2a; methylation at the PPARγ promoter	[137,138]
	↑SET7	Increase in TNFa, CCL2 and IL-8 production	[139]
	↑ SETDB1	Decrease in TNFa production	[140]
	↑SETDB2	Negative regulation of CXCL1, IL-12b, CXCL2, YM1 production	[141]
	↑SUV39H2	Decrease in IL-6 and TNFa	[142]
	↑SUV40H1	Positive regulation of TNF and CXCL10 production	[143]
	↑SUV40H2		
	↑SMYD2	Negative regulation of TNF, IL-6, MHC-II, CD40/80	[144]
	↑SMYD5	Associated with low production of TNF, IL-1a, IL-1b, CCL4, CXCL10	[143]
	↑ASH1	Negative regulation of IL-6, TNFa production	[145]
	↑MLL1	Increase in CXCL10 level	[146]
	↑MLL4	Associated with activation of LPS signaling via Pigp	[147]
	↑EZH1	Negative regulation of TLR signaling and production of IL-6, TNF, IFNb	[148]
	↑EZH2	Associated with decrease in CCL2, CCL8 expression	[149]
	↑EHMT2(G9A)	Increase in LPS tolerance	[150,151,152]
HDM (histone demethylases)	↑JMJD2	Down-regulation of IL-12b and IFNb production	[153]
	↑JMJD3	Activation of TNFa production; Associated with low level of ARG1, YM1, IRF4, FIZZ1 and CD206	[31,154,155]
	↑ UTX	Increase in IL-6, INFb	[156]
	↑AOF1	Positive regulation of NFκB signaling; Increase in CCL22, IL-12b production	[157]
HDAC (histone deacetylases)	↑SIRT1	Inhibition of NFκB signaling, decrease in CCL2, IL-1b, IL-6, NOS2 and TNFa level	[150,158,159]
	↑SIRT2	Negative regulation of NFκB signaling, decrease in TNFa, IL-6, CCL2, IL-1b production; Increase in GATA3, ARG1, CD11c expression	[160]
	↓SIRT1/SIRT2	Increase in IL-1a, IL-1b, IL-10 and TNFa production	[161]
	↑SIRT6	Decrease in IL-1b production	[152]
	↑HDAC1	Positive regulation of IFN signaling, IRF3 activation and decrease in IL-6 production	[162,163]
	↑HDAC2	Decrease in IL-6, MHC-II level, activation of IFN signaling and IRF3 production	[136,162,164]
	↑HDAC3	Activation of IL-6, NO, IFNβ, NOS2 production, decrease in TGFb level; Attenuates IL-4 signaling	[15,121,165,166]
	↑HDAC4	Increase in TNF, IL-6 level; Activation of STAT6 signaling, associated with expression of ARG1	[167,168]
	↑HDAC5	Increase in TNF, CCL2, IL-10 production	[169]
	↑HDAC6	Associated with LPS activation; decrease in ROS production	[170,171]
	↑HDAC7	Activation of TLR signaling	[66]
	↑HDAC11	Activation of antigen presentation, CD4+ T cell stimulation; decrease in IL-10 and IL-1b production	[172,173]
BET (bromodomain and extraterminal proteins)	↑BET	Increase in production of IFNg, IL-1b, IL-1a, IL-6, IL-12b, CCL5, CXCL10, CXCL2/3	[174,175]

Notes: ARG1—Arginase 1; LPS—lipopolysaccharide; ROS—reactive oxygen species; TLR—Toll-like receptor.

**Table 3:** Mechanisms of epigenetic regulation in tumor-associated macrophages (TAMs).

Epigenetic Process	The Functional Association with TAMs	Activated TAM Function
DNA methylation	TET2 expression is increased in the intratumoral myeloid cells both in murine model of melanoma and patients with melanoma	Immunosuppression [135]
Histone modification	ERK-1/2-dependent histone phosphorylation on the IL-10 promoter results in the increase in the IL-10 production. The activation of ERK-1/2 leads to the inhibition of MyD88 via IRAK M, disrupting TLR-signaling in TAMs in murine melanoma mode	Immunosuppression [190]
	BRD4 is involved in the IL-4-controlled transcriptional programming of macrophages and TAM-mediated immunosuppression in TME in murine models for lung cancer, colon cancer and melanoma	Immunosuppression [191]
	DcR3 inhibits the expression of HLA-DR via the histone deacetylation on the CIITA promoters in human TAMs generated in vitro by monocytes stimulation with colorectal cancer cell conditioned medium	Immunosuppression [192]
	LSD1 demethylase can regulate genes associated with M2 –polarization by activating the production of checkpoint mediators (NOS2, Gpr18, IL-8, IL-1b, IL-12b, CCR7, Fpr2) in a murine model for triple negative breast cancer	Immunosuppression [98,193]
	Pan HDAC inhibition activates M2-like function in human monocyte-derived macrophages (in vitro) by indcution of secretion of IL-10 and VEGF-A and induces the EMT in prostate cancer cells	Immunosuppression, angiogenesis [194]
	Inhibition of HDAC by SAHA results in a decrease in NO production; SAHA can decrease TAM infiltration into the mammary gland in PyMT mice and thereafter decrease the tumor size in breast, lung and pancreatic cancer mouse model	NO production, migration [195]
	Jumonji domain-containing HDM1A enhances TAM infiltration and angiogenic activity in SCID mice injected with human HeLa and A673 cells through upregulating pro-angiogenic factors such as angiopoietins and FGFs	Migration, angiogenesis [196]
miRNA	Exosomal miR-1246 and miR-21 can increase IL-10, TGF-β, and MMP production in primary human macrophagesin vitro	Activation of M2 polarization, migration [197]
	In EBV-negative diffuse large B-cell lymphoma overexpression of miR-155 has negative correlation with differentiation toward M2. In EBV + diffuse large B-cell lymphoma patients, positive correlation between miR-155 relative expression and CD163/CD68 ratio was found	Activation of M2 program [198]
	miR-155 regulates the production of TNFa, IL-6 and IL-10, and negative correlates with the expression of C/EBP in human cells stimulated by cancer cell line (cervical, hepatocellular) conditioned media	Cytokine production [199]
	miR-17 and miR-20a regulate IL6-dependent HIF2a activation in monocytes that have been incubated with Hep2 condition media, and in CD14+ cells from patients with HCC	Angiogenesis [200]
	Increased expression of miR-511-3p suppresses tumor supporting CD206+ TAM gene signatures in murine model with injected LLC	Inhibition of M2 program [201]
	miR-19a-3p is capable for the inhibition of the M2 phenotype trough the upregulation of Fra-1 and the Fra-1/STAT3 signaling pathway in murine model for breast cancer	Inhibition of M2 program [202]

Notes: BRD4—bromodomain-containing protein 4; DcR3—decoy receptor 3; EBV—Epstein-Barr virus; EMT—epithelial-mesenchymal transition; FGF—fibroblast growth factor; HCC—hepatocellular carcinoma; HDAC—Histone deacetylase; HDM1A—Histone demethylase 1A; JMJD1A—jumonji domain-containing protein 1A; LLC—Lewis lung carcinoma; LSD1—lysine-specific histone demethylase 1; MMP—matrix metalloproteinase; SCID—severe combined immunodeficiency; TET2—Tet methylcytosine dioxygenase.

**Table 4:** TAM reprogramming by tumor-derived metabolic factors.

Metabolic Pathway	Metabolic Factor in TME	Function of Macrophages in TME	Experimental Model of TAMs	References
Glycolysis	Tumor-derived lactate	Upregulation of CD206 and CD163 expression and elevated production of TGF-b1, IL-10, VEGF. Increased secretion of CCL5 by TAMs which induce cancer cell migration, enhanced EMT and aerobic glycolysis in breast cancer cells	Co-culture system of human monocytic cell line THP-1 with human breast cancer MDA-MB-231 cells	[244]
Glycolysis	Tumor-derived condition medium	Up-regulation of a number of glycolytic genes, including HK2, GPI, ALDOA, TPI1 and PGK1, in macrophages. Macrophage-mediated activation of vascular network formation, increased extravasation of tumor cells out of blood vessels and EMT in tumor cells	Human monocytes differentiated in the presence of condition medium from PDAC cells	[245]
Glycolysis	Tumor-derived condition medium	Increase in glycolytic enzymes—HK2, ENO1, and PFKL	TAMs from MMTV-PyMT mice and BMDMs stimulated by tumor extract from MMTV-PyMT mice	[246]
Glycolysis	Tumor-derived condition medium	Increased glycolysis (high GLUT1 and HK2 expression), and high amounts of lactate via Akt–mTOR-dependent pathway, in parallel with activated OXPHOS and reduced FAO	Human blood monocytes stimulated with the conditioned medium of melanoma cells (MV3)	[247]
Glycolysis	Tumor-derived condition medium	Increased glycolysis in human macrophages by elevation of ECAR in mTOR-dependent manner. Increase in glycolytic enzymes and lactate receptor (GBR18, PFKFB3, PKM2) in TAMs of patient specimens	Human macrophages stimulated with TC-conditioned medium and tumor samples from patients with TC	[248]
Glycolysis	Hypoxia	Inhibition of mTORC1 by REDD1 shift macrophages toward the immunosuppressive and pro-angiogenic phenotype, by inhibition of glucose uptake and glycolysis and enhancing glucose availability for endothelial cells	TAMs from MMTV-PyMT mice	[249]
Glycolysis	Combination of hypoxia and tumor-derived lactate	Induction of ARG1 and VEGF-A	TAMs from MMTV-PyMT mice	[250,251]
	Hypoxia	The accumulation of M2-macrophages with elevated expression of YM1, FIZZ1, IL-10	TAMs from murine B16 melanoma cells-derived tumor	[252]
Glycolysis	ROS produced by cancer cells and NOX4-overexpressed tumor cells	ROS stimulates production, of CCL7, IL8, CSF-1 and VEGF-C, that contribute to enhanced NSCLC cell growth. NOX4-overexpressed tumor cells enhanced the recruitment of M2-like TAMs via ROS/PI3K signaling-dependent pathway	Co-culture of macrophages with lung cancer cell lines A549 and Calu-1	[253]
	Tumor-derived hypoxia	Strong chemotactic effect toward THP-1 cells, upregulation of M2 marker expression (IL-10 and CCL-22) and downregulation of M1 markers (IL-6 and TNF-α)	THP-1 stimulated with supernatans from U87 or U251 glioma cell	[254]
OXPHOS	Hypoxia	The recruitment and differentiation of F4/80+CD206+ population. MFs exhibited enhanced OXPHOS activity inhibiting AKT and mTOR	BMDMs treated with exosomes derived from hypoxic B16-F0, A375, A431, and A549 lung adenocarcinoma cells	[255]
OXPHOS	Tumor-derived factors	GS-dependent increased expression of M2-specific markers (ARG1, CD206, CCL17, and CCL22) and IL10 production via the mTOR signaling	TAMs from mice implanted with LLC cells	[256]
FAO	Oleate	Lipid droplet-dependent up-regulation of M2 specific markers (CD206, IL-6, VEGFα, MMP9, ARG1) and induction of immunosuppressive TAMs by supporting mitochondrial respiration	TAMs from murine colon carcinoma model in vivo	[257]
FAO		E-FABP-dependent lipid droplet formation and activation of M1-like (MHCII+CD11c+), significant inhibition of tumor growth	Mouse mammary adenocarcinoma model	[258]
FAO	Tumor-derived factors	Increased production of proinflammatory chemokine CCL2 and immunosuppressive cytokine IL-10, increased metabolism of arachidonic acid	TAMs isolated from human renal cell carcinoma	[259]
FAO	Tumor-derived itaconic acid	Enhanced OXPHOS and ROS production in TAMs. Increased tumor burdan	TAMs from tumor-bearing mice (B16 melanoma and ID8 ovarian carcinoma)	[260]

Notes: BMDMs—bone marrow-derived macrophages; ECAR—extracellular acidification rate; EMT—epithelial-mesenchymal transition; FAO—fatty acid oxidation; GS—glutamate synthesis; LLC—Lewis lung carcinoma; NSCLC—non-small-cell lung carcinoma; OXPHOS—oxidative phosphorylation; PDAC—pancreatic ductal adenocarcinoma; TC—thyroid cancer.

## Abbreviations

ARG1	Arginase 1
BETs	Bromodomain and extraterminal proteins
BHA	Butylated hydroxyanisole
BMDMs	Bone marrow-derived macrophage
DFS	Disease-free survival
DNMTs	DNA methyltransferases
ECARs	Extracellular acidification rates
ECM	Extracellular matrix
EMT	Epithelial–mesenchymal transition
ETC	Electron transport chain
FAS	Fatty acid synthesis
FAO	Fatty acid oxidation
GLUT1	Glucose transporter
GS	Glutamine-synthetase
HATs	Histone acetyltransferases
HDACs	Histone deacetylases
HDMs	Histone demethylases
HIF-1α	Hypoxia-inducible factor 1α
HK2	Hexokinase
HMTs	Histone methyltransferases
IHC	Immunohistochemical analysis
IRFs	Interferon regulatory factors
LDs	Lipid droplets
LDHA	Lactate dehydrogenase A
LDL	Low density lipoproteins
LPS	Lipopolysaccharide
MDSCs	Myeloid-derived suppressor cell
mTORC1	Mammalian target of rapamycin complex 1
mTORC2	Mammalian target of rapamycin complex 2
NLRP3	NOD-, LRR- and pyrin domain-containing protein 3
NO	Nitrogen intermediates
OCR	Oxygen consumption rate
OS	Overall survival
PDH	Pyruvate dehydrogenase
PDK1	Pyruvate dehydrogenase kinase 1
PKM2	Pyruvate kinase M2
PPP	Pentose phosphate pathway
RAW264.7	Mouse alveolar macrophage cell line
ROS	Reactive oxygen species
TAMs	Tumor-associated macrophages
TCA	Tricarboxylic acid
TET	Ten-eleven translocation proteins
TFs	Transcription factors
TLR	Toll-like receptor
TME	Tumor microenvironment

## References

[1] Stakheyeva M., Riabov V., Mitrofanova I., Litviakov N., Choynzonov E., Cherdyntseva N., Kzhyshkowska J. (2017). Role of the immune component of tumor microenvironment in the efficiency of cancer treatment: Perspectives for the personalized therapy. Curr. Pharm. Des..

[2] Vitale I., Manic G., Coussens L.M., Kroemer G., Galluzzi L. (2019). Macrophages and metabolism in the tumor microenvironment. Cell Metab..

[3] Wang M., Zhao J., Zhang L., Wei F., Lian Y., Wu Y., Gong Z., Zhang S., Zhou J., Cao K. (2017). Role of tumor microenvironment in tumorigenesis. J. Cancer.

[4] Kzhyshkowska J., Grigoryeva E., Larionova I., Bizzarri M. (2020). Targeting the Tumor-Associated Macrophages for ‘Normalizing’ Cancer. Approaching Complex. Diseases.

[5] Riabov V., Gudima A., Wang N., Orekhov A., Mickley A., Kzhyshkowska J. (2014). Role of tumor associated macrophages in tumor angiogenesis and lymphangiogenesis. Front. Physiol..

[6] Kzhyshkowska J., Larionova I., Liu T. (2019). YKL-39 as a potential new target for anti-angiogenic therapy in cancer. Front. Immunol..

[7] Mitrofanova I., Zavyalova M., Riabov V., Cherdyntseva N., Kzhyshkowska J. (2018). The effect of neoadjuvant chemotherapy on the correlation of tumor-associated macrophages with CD31 and LYVE-1. Immunobiology.

[8] Sica A., Mantovani A. (2012). Macrophage plasticity and polarization: In vivo veritas. J. Clin. Investig..

[9] Larionova I., Cherdyntseva N., Liu T., Patysheva M., Rakina M., Kzhyshkowska J. (2019). Interaction of tumor-associated macrophages and cancer chemotherapy. Oncoimmunology.

[10] Viola A., Munari F., Sánchez-Rodríguez R., Scolaro T., Castegna A. (2019). The metabolic signature of macrophage responses. Front. Immunol..

[11] Thapa B., Lee K. (2019). Metabolic influence on macrophage polarization and pathogenesis. Bmb Rep..

[12] Tugal D., Liao X., Jain M.K. (2013). Transcriptional control of macrophage polarization. Arterioscler. Thromb. Vasc. Biol..

[13] Lawrence T., Natoli G. (2011). Transcriptional regulation of macrophage polarization: Enabling diversity with identity. Nat. Rev. Immunol..

[14] Li H., Jiang T., Li M.-Q., Zheng X.-L., Zhao G.-J. (2018). Transcriptional regulation of macrophages polarization by MicroRNAs. Front. Immunol..

[15] Hoeksema M.A., de Winther M.P. (2016). Epigenetic regulation of monocyte and macrophage function. Antioxid. Redox Signal..

[16] Hume D.A., Freeman T.C. (2014). Transcriptomic analysis of mononuclear phagocyte differentiation and activation. Immunol. Rev..

[17] Jaiswal A., Reddy S.S., Maurya M., Maurya P., Barthwal M.K. (2019). MicroRNA-99a mimics inhibit M1 macrophage phenotype and adipose tissue inflammation by targeting TNFα. Cell. Mol. Immunol..

[18] Xu H., Zhu J., Smith S., Foldi J., Zhao B., Chung A.Y., Outtz H., Kitajewski J., Shi C., Weber S. (2012). Notch–RBP-J signaling regulates the transcription factor IRF8 to promote inflammatory macrophage polarization. Nat. Immunol..

[19] DeKoter R.P., Singh H. (2000). Regulation of B lymphocyte and macrophage development by graded expression of PU. 1. Science.

[20] Jablonski K.A., Amici S.A., Webb L.M., de Dios Ruiz-Rosado J., Popovich P.G., Partida-Sanchez S., Guerau-de-Arellano M. (2015). Novel markers to delineate murine M1 and M2 macrophages. PLoS ONE.

[21] Pello O.M., De Pizzol M., Mirolo M., Soucek L., Zammataro L., Amabile A., Doni A., Nebuloni M., Swigart L.B., Evan G.I. (2012). Role of c-MYC in alternative activation of human macrophages and tumor-associated macrophage biology. Blood.

[22] Yang Y., Ye Y.-C., Chen Y., Zhao J.-L., Gao C.-C., Han H., Liu W.-C., Qin H.-Y. (2018). Crosstalk between hepatic tumor cells and macrophages via Wnt/β-catenin signaling promotes M2-like macrophage polarization and reinforces tumor malignant behaviors. Cell Death Dis..

[23] Pello O.M., Chevre R., Laoui D., De Juan A., Lolo F., Andrés-Manzano M.J., Serrano M., Van Ginderachter J.A., Andres V. (2012). In vivo inhibition of c-MYC in myeloid cells impairs tumor-associated macrophage maturation and pro-tumoral activities. PLoS ONE.

[24] Jeyakumar T., Fodil N., Van Der Kraak L., Meunier C., Cayrol R., McGregor K., Langlais D., Greenwood C.M., Beauchemin N., Gros P. (2019). Inactivation of interferon Regulatory Factor 1 Causes Susceptibility to Colitis-Associated colorectal cancer. Sci. Rep..

[25] Chistiakov D.A., Myasoedova V.A., Revin V.V., Orekhov A.N., Bobryshev Y.V. (2018). The impact of interferon-regulatory factors to macrophage differentiation and polarization into M1 and M2. Immunobiology.

[26] Tarassishin L., Suh H.-S., Lee S.C. (2011). Interferon regulatory factor 3 plays an anti-inflammatory role in microglia by activating the PI3K/Akt pathway. J. Neuroinflammation.

[27] Biswas S.K., Gangi L., Paul S., Schioppa T., Saccani A., Sironi M., Bottazzi B., Doni A., Vincenzo B., Pasqualini F. (2006). A distinct and unique transcriptional program expressed by tumor-associated macrophages (defective NF-κB and enhanced IRF-3/STAT1 activation). Blood.

[28] Zhuang H., Dai X., Zhang X., Mao Z., Huang H. (2020). Sophoridine suppresses macrophage-mediated immunosuppression through TLR4/IRF3 pathway and subsequently upregulates CD8+ T cytotoxic function against gastric cancer. Biomed. Pharmacother..

[29] Liu B., Wang X., Chen T.-Z., Li G.-L., Tan C.-C., Chen Y., Duan S.-Q. (2016). Polarization of M1 tumor associated macrophage promoted by the activation of TLR3 signal pathway. Asian Pac. J. Trop. Med..

[30] Romieu-Mourez R., Solis M., Nardin A., Goubau D., Baron-Bodo V., Lin R., Massie B., Salcedo M., Hiscott J. (2006). Distinct roles for IFN regulatory factor (IRF)-3 and IRF-7 in the activation of antitumor properties of human macrophages. Cancer Res..

[31] Satoh T., Takeuchi O., Vandenbon A., Yasuda K., Tanaka Y., Kumagai Y., Miyake T., Matsushita K., Okazaki T., Saitoh T. (2010). The Jmjd3-Irf4 axis regulates M2 macrophage polarization and host responses against helminth infection. Nat. Immunol..

[32] Su C.Y., Fu X.L., Duan W., Yu P.W., Zhao Y.L. (2018). High density of CD68+ tumor-associated macrophages predicts a poor prognosis in gastric cancer mediated by IL-6 expression. Oncol. Lett..

[33] Zhang F., Parayath N., Ene C., Stephan S., Koehne A., Coon M., Holland E., Stephan M. (2019). Genetic programming of macrophages to perform anti-tumor functions using targeted mRNA nanocarriers. Nat. Commun..

[34] Krausgruber T., Blazek K., Smallie T., Alzabin S., Lockstone H., Sahgal N., Hussell T., Feldmann M., Udalova I.A. (2011). IRF5 promotes inflammatory macrophage polarization and T H 1-T H 17 responses. Nat. Immunol..

[35] Pinilla-Vera M., Xiong Z., Zhao Y., Zhao J., Donahoe M.P., Barge S., Horne W.T., Kolls J.K., McVerry B.J., Birukova A. (2016). Full spectrum of LPS activation in alveolar macrophages of healthy volunteers by whole transcriptomic profiling. PLoS ONE.

[36] Twum D.Y., Colligan S.H., Hoffend N.C., Katsuta E., Gomez E.C., Hensen M.L., Seshadri M., Nemeth M.J., Abrams S.I. (2019). IFN regulatory factor–8 expression in macrophages governs an antimetastatic program. Jci Insight.

[37] Muhitch J.B., Hoffend N.C., Azabdaftari G., Miller A., Bshara W., Morrison C.D., Schwaab T., Abrams S.I. (2019). Tumor-associated macrophage expression of interferon regulatory Factor-8 (IRF8) is a predictor of progression and patient survival in renal cell carcinoma. J. Immunother. Cancer.

[38] Liao X., Sharma N., Kapadia F., Zhou G., Lu Y., Hong H., Paruchuri K., Mahabeleshwar G.H., Dalmas E., Venteclef N. (2011). Krüppel-like factor 4 regulates macrophage polarization. J. Clin. Investig..

[39] Kapoor N., Niu J., Saad Y., Kumar S., Sirakova T., Becerra E., Li X., Kolattukudy P.E. (2015). Transcription factors STAT6 and KLF4 implement macrophage polarization via the dual catalytic powers of MCPIP. J. Immunol..

[40] Date D., Das R., Narla G., Simon D.I., Jain M.K., Mahabeleshwar G.H. (2014). Kruppel-like transcription factor 6 regulates inflammatory macrophage polarization. J. Biol. Chem..

[41] Goodman W.A., Omenetti S., Date D., Di Martino L., De Salvo C., Kim G.-D., Chowdhry S., Bamias G., Cominelli F., Pizarro T.T. (2016). KLF6 contributes to myeloid cell plasticity in the pathogenesis of intestinal inflammation. Mucosal Immunol..

[42] Gemelli C., Marani T.Z., Bicciato S., Mazza E.M., Boraschi D., Salsi V., Zappavigna V., Parenti S., Selmi T., Tagliafico E. (2014). MafB is a downstream target of the IL-10/STAT3 signaling pathway, involved in the regulation of macrophage de-activation. Biochim. Et Biophys. Acta (Bba)-Mol. Cell Res..

[43] Yadav M.K., Inoue Y., Nakane-Otani A., Tsunakawa Y., Jeon H., Samir O., Teramoto A., Kulathunga K., Kusakabe M., Nakamura M. (2020). Transcription factor MafB is a marker of tumor-associated macrophages in both mouse and humans. Biochem. Biophys. Res. Commun..

[44] Cuevas V.D., Anta L., Samaniego R., Orta-Zavalza E., de la Rosa J.V., Baujat G., Domínguez-Soto Á., Sánchez-Mateos P., Escribese M.M., Castrillo A. (2017). MAFB determines human macrophage anti-inflammatory polarization: Relevance for the pathogenic mechanisms operating in multicentric carpotarsal osteolysis. J. Immunol..

[45] Tellechea M., Buxadé M., Tejedor S., Aramburu J., López-Rodríguez C. (2018). NFAT5-regulated macrophage polarization supports the proinflammatory function of macrophages and T lymphocytes. J. Immunol..

[46] Hagemann T., Lawrence T., McNeish I., Charles K.A., Kulbe H., Thompson R.G., Robinson S.C., Balkwill F.R. (2008). “Re-educating” tumor-associated macrophages by targeting NF-κB. J. Exp. Med..

[47] Shen J., Sun X., Pan B., Cao S., Cao J., Che D., Liu F., Zhang S., Yu Y. (2018). IL-17 induces macrophages to M2-like phenotype via NF-κB. Cancer Manag. Res..

[48] Qian F., Deng J., Lee Y.G., Zhu J., Karpurapu M., Chung S., Zheng J.-N., Xiao L., Park G.Y., Christman J.W. (2015). The transcription factor PU. 1 promotes alternative macrophage polarization and asthmatic airway inflammation. J. Mol. Cell Biol..

[49] Yashiro T., Nakano S., Nomura K., Uchida Y., Kasakura K., Nishiyama C. (2019). A transcription factor PU. 1 is critical for Ccl22 gene expression in dendritic cells and macrophages. Sci. Rep..

[50] Bi J., Zeng X., Zhao L., Wei Q., Yu L., Wang X., Yu Z., Cao Y., Shan F., Wei M. (2016). miR-181a induces macrophage polarized to M2 phenotype and promotes M2 macrophage-mediated tumor cell metastasis by targeting KLF6 and C/EBPα. Mol. Ther. Nucleic Acids.

[51] Karpurapu M., Wang X., Deng J., Park H., Xiao L., Sadikot R.T., Frey R.S., Maus U.A., Park G.Y., Scott E.W. (2011). Functional PU. 1 in macrophages has a pivotal role in NF-κB activation and neutrophilic lung inflammation during endotoxemia. Blood J. Am. Soc. Hematol..

[52] Ha S.-D., Cho W., DeKoter R.P., Kim S.O. (2019). The transcription factor PU. 1 mediates enhancer-promoter looping that is required for IL-1β eRNA and mRNA transcription in mouse melanoma and macrophage cell lines. J. Biol. Chem..

[53] Zhang F., Wang H., Wang X., Jiang G., Liu H., Zhang G., Wang H., Fang R., Bu X., Cai S. (2016). TGF-β induces M2-like macrophage polarization via SNAIL-mediated suppression of a pro-inflammatory phenotype. Oncotarget.

[54] Ma B., Yang Y., Li Z., Zhao D., Zhang W., Jiang Y., Xue D. (2018). Modular bioinformatics analysis demonstrates that a Toll-like receptor signaling pathway is involved in the regulation of macrophage polarization. Mol. Med. Rep..

[55] Petrillo M., Zannoni G.F., Martinelli E., Anchora L.P., Ferrandina G., Tropeano G., Fagotti A., Scambia G. (2015). Polarisation of tumor-associated macrophages toward M2 phenotype correlates with poor response to chemoradiation and reduced survival in patients with locally advanced cervical cancer. PLoS ONE.

[56] Kusmartsev S., Gabrilovich D.I. (2005). STAT1 signaling regulates tumor-associated macrophage-mediated T cell deletion. J. Immunol..

[57] Solís-Martínez R., Cancino-Marentes M., Hernández-Flores G., Ortiz-Lazareno P., Mandujano-Álvarez G., Cruz-Gálvez C., Sierra-Díaz E., Rodríguez-Padilla C., Jave-Suárez L., Aguilar-Lemarroy A. (2018). Regulation of immunophenotype modulation of monocytes-macrophages from M1 into M2 by prostate cancer cell-culture supernatant via transcription factor STAT3. Immunol. Lett..

[58] Feng Y., Ren J., Gui Y., Wei W., Shu B., Lu Q., Xue X., Sun X., He W., Yang J. (2018). Wnt/β-catenin–promoted macrophage alternative activation contributes to kidney fibrosis. J. Am. Soc. Nephrol..

[59] Yin Z., Ma T., Lin Y., Lu X., Zhang C., Chen S., Jian Z. (2018). IL6/STAT3 pathway intermediates M1/M2 macrophage polarization during the development of hepatocellular carcinoma. J. Cell. Biochem..

[60] Kujawski M., Kortylewski M., Lee H., Herrmann A., Kay H., Yu H. (2008). Stat3 mediates myeloid cell–dependent tumor angiogenesis in mice. J. Clin. Investig..

[61] Yu T., Gan S., Zhu Q., Dai D., Li N., Wang H., Chen X., Hou D., Wang Y., Pan Q. (2019). Modulation of M2 macrophage polarization by the crosstalk between Stat6 and Trim24. Nat. Commun..

[62] Fang L., Hodge J., Saaoud F., Wang J., Iwanowycz S., Wang Y., Hui Y., Evans T.D., Razani B., Fan D. (2017). Transcriptional factor EB regulates macrophage polarization in the tumor microenvironment. OncoImmunology.

[63] Shibata Y., Berclaz P.-Y., Chroneos Z.C., Yoshida M., Whitsett J.A., Trapnell B.C. (2001). GM-CSF regulates alveolar macrophage differentiation and innate immunity in the lung through PU. 1. Immunity.

[64] Bonfield T.L., Raychaudhuri B., Malur A., Abraham S., Trapnell B.C., Kavuru M.S., Thomassen M.J. (2003). PU. 1 regulation of human alveolar macrophage differentiation requires granulocyte-macrophage colony-stimulating factor. Am. J. Physiol. -Lung Cell. Mol. Physiol..

[65] Rojo R., Pridans C., Langlais D., Hume D.A. (2017). Transcriptional mechanisms that control expression of the macrophage colony-stimulating factor receptor locus. Clin. Sci..

[66] Shakespear M.R., Hohenhaus D.M., Kelly G.M., Kamal N.A., Gupta P., Labzin L.I., Schroder K., Garceau V., Barbero S., Iyer A. (2013). Histone deacetylase 7 promotes Toll-like receptor 4-dependent proinflammatory gene expression in macrophages. J. Biol. Chem..

[67] Lin J., Liu W., Luan T., Yuan L., Jiang W., Cai H., Yuan W., Wang Y., Zhang Q., Wang L. (2017). High expression of PU. 1 is associated with Her-2 and shorter survival in patients with breast cancer. Oncol. Lett..

[68] Ueno N., Nishimura N., Ueno S., Endo S., Tatetsu H., Hirata S., Hata H., Matsuoka M., Mitsuya H., Okuno Y. (2017). PU. 1 acts as tumor suppressor for myeloma cells through direct transcriptional repression of IRF4. Oncogene.

[69] Antony-Debré I., Paul A., Leite J., Mitchell K., Kim H.M., Carvajal L.A., Todorova T.I., Huang K., Kumar A., Farahat A.A. (2017). Pharmacological inhibition of the transcription factor PU. 1 in leukemia. J. Clin. Investig..

[70] Xu Y., Gu S., Bi Y., Qi X., Yan Y., Lou M. (2018). Transcription factor PU. 1 is involved in the progression of glioma. Oncol. Lett..

[71] Song L.-J., Zhang W.-J., Chang Z.-W., Pan Y.-F., Zong H., Fan Q.-X., Wang L.-X. (2015). PU. 1 is identified as a novel metastasis suppressor in hepatocellular carcinoma regulating the miR-615-5p/IGF2 axis. Asian Pac. J. Cancer Prev..

[72] Shen C., Chen M.-T., Zhang X.-H., Yin X.-L., Ning H.-M., Su R., Lin H.-S., Song L., Wang F., Ma Y.-N. (2016). The PU. 1-modulated microRNA-22 is a regulator of monocyte/macrophage differentiation and acute myeloid leukemia. PLoS Genet..

[73] Tamma R., Ingravallo G., Gaudio F., Annese T., Albano F., Ruggieri S., Dicataldo M., Maiorano E., Specchia G., Ribatti D. (2020). STAT3, tumor microenvironment, and microvessel density in diffuse large B cell lymphomas. Leuk. Lymphoma.

[74] Yan D., Wang H.-W., Bowman R.L., Joyce J.A. (2016). STAT3 and STAT6 signaling pathways synergize to promote cathepsin secretion from macrophages via IRE1α activation. Cell Rep..

[75] Goenka S., Kaplan M.H. (2011). Transcriptional regulation by STAT6. Immunol. Res..

[76] Chen W., Xu Y., Zhong J., Wang H., Weng M., Cheng Q., Wu Q., Sun Z., Jiang H., Zhu M. (2016). MFHAS1 promotes colorectal cancer progress by regulating polarization of tumor-associated macrophages via STAT6 signaling pathway. Oncotarget.

[77] Lee C.-C., Lin J.-C., Hwang W.-L., Kuo Y.-J., Chen H.-K., Tai S.-K., Lin C.-C., Yang M.-H. (2018). Macrophage-secreted interleukin-35 regulates cancer cell plasticity to facilitate metastatic colonization. Nat. Commun..

[78] Andersen M.N., Etzerodt A., Graversen J.H., Holthof L.C., Moestrup S.K., Hokland M., Møller H.J. (2019). STAT3 inhibition specifically in human monocytes and macrophages by CD163-targeted corosolic acid-containing liposomes. Cancer Immunol. Immunother..

[79] Li Q., Hao Z., Hong Y., He W., Zhao W. (2018). Reprogramming tumor associated macrophage phenotype by a polysaccharide from ilex asprella for sarcoma immunotherapy. Int. J. Mol. Sci..

[80] Giuliani C., Bucci I., Napolitano G. (2018). The role of the transcription factor Nuclear Factor-kappa B in thyroid autoimmunity and cancer. Front. Endocrinol..

[81] Shrihari T. (2017). Dual role of inflammatory mediators in cancer. Ecancermedicalscience.

[82] Szebeni G.J., Vizler C., Kitajka K., Puskas L.G. (2017). Inflammation and cancer: Extra-and intracellular determinants of tumor-associated macrophages as tumor promoters. Mediat. Inflamm..

[83] Bally A.P., Lu P., Tang Y., Austin J.W., Scharer C.D., Ahmed R., Boss J.M. (2015). NF-κB regulates PD-1 expression in macrophages. J. Immunol..

[84] Liu C.-P., Zhang X., Tan Q.-L., Xu W.-X., Zhou C.-Y., Luo M., Li X., Huang R.-Y., Zeng X. (2017). NF-κB pathways are involved in M1 polarization of RAW 264.7 macrophage by polyporus polysaccharide in the tumor microenvironment. PLoS ONE.

[85] Sica A., Saccani A., Bottazzi B., Polentarutti N., Vecchi A., Van Damme J., Mantovani A. (2000). Autocrine production of IL-10 mediates defective IL-12 production and NF-κB activation in tumor-associated macrophages. J. Immunol..

[86] Hayward W.S., Neel B.G., Astrin S.M. (1981). Activation of a cellular onc gene by promoter insertion in ALV-induced lymphoid leukosis. Nature.

[87] Adhikary S., Eilers M. (2005). Transcriptional regulation and transformation by Myc proteins. Nat. Rev. Mol. Cell Biol..

[88] Li L., Ng D., Mah W., Almeida F., Rahmat S., Rao V., Leow S., Laudisi F., Peh M., Goh A. (2015). A unique role for p53 in the regulation of M2 macrophage polarization. Cell Death Differ..

[89] Wang Q., Cheng F., Ma T.-t., Xiong H.-Y., Li Z.-W., Xie C.-L., Liu C.-Y., Tu Z.-G. (2016). Interleukin-12 inhibits the hepatocellular carcinoma growth by inducing macrophage polarization to the M1-like phenotype through downregulation of Stat-3. Mol. Cell. Biochem..

[90] Mancino A., Natoli G. (2016). Specificity and function of IRF family transcription factors: Insights from genomics. J. Interferon Cytokine Res..

[91] Duan Y., Li Z., Cheng S., Chen Y., Zhang L., He J., Liao Q., Yang L., Gong Z., Sun L.-Q. (2015). Nasopharyngeal carcinoma progression is mediated by EBER-triggered inflammation via the RIG-I pathway. Cancer Lett..

[92] Sugiyama Y., Kakoi K., Kimura A., Takada I., Kashiwagi I., Wakabayashi Y., Morita R., Nomura M., Yoshimura A. (2012). Smad2 and Smad3 are redundantly essential for the suppression of iNOS synthesis in macrophages by regulating IRF3 and STAT1 pathways. Int. Immunol..

[93] Zhao J.-L., Huang F., He F., Gao C.-C., Liang S.-Q., Ma P.-F., Dong G.-Y., Han H., Qin H.-Y. (2016). Forced activation of Notch in macrophages represses tumor growth by upregulating miR-125a and disabling tumor-associated macrophages. Cancer Res..

[94] Ma S., Liu M., Xu Z., Li Y., Guo H., Ge Y., Liu Y., Zheng D., Shi J. (2016). A double feedback loop mediated by microRNA-23a/27a/24-2 regulates M1 versus M2 macrophage polarization and thus regulates cancer progression. Oncotarget.

[95] Dannenmann S.R., Thielicke J., Stöckli M., Matter C., Von Boehmer L., Cecconi V., Hermanns T., Hefermehl L., Schraml P., Moch H. (2013). Tumor-associated macrophages subvert T-cell function and correlate with reduced survival in clear cell renal cell carcinoma. OncoImmunology.

[96] Langlais D., Barreiro L.B., Gros P. (2016). The macrophage IRF8/IRF1 regulome is required for protection against infections and is associated with chronic inflammation. J. Exp. Med..

[97] Guo Y., Yang Z., Wu S., Xu P., Peng Y., Yao M. (2017). Inhibition of IRF8 negatively regulates macrophage function and impairs cutaneous wound healing. Inflammation.

[98] Hsieh C.-H., Tai S.-K., Yang M.-H. (2018). Snail-overexpressing cancer cells promote M2-like polarization of tumor-associated macrophages by delivering MiR-21-abundant exosomes. Neoplasia.

[99] Pioli P.D. (2015). Modulation of Lymphocyte Development and Function via Snail Family Transcriptional Regulators.

[100] Tan A.H.Y., Tu W., McCuaig R.D., Hardy K., Donovan T., Tsimbalyuk S., Forwood J.K., Rao S. (2019). Lysine-specific histone demethylase 1A regulates macrophage polarization and checkpoint molecules in the tumor microenvironment of triple-negative breast cancer. Front. Immunol..

[101] Eychène A., Rocques N., Pouponnot C. (2008). A new MAF ia in cancer. Nat. Rev. Cancer.

[102] Daassi D., Hamada M., Jeon H., Imamura Y., Tran M.T.N., Takahashi S. (2016). Differential expression patterns of MafB and c-Maf in macrophages in vivo and in vitro. Biochem. Biophys. Res. Commun..

[103] Kim H. (2017). The transcription factor MafB promotes anti-inflammatory M2 polarization and cholesterol efflux in macrophages. Sci. Rep..

[104] Hasegawa H., Watanabe T., Kato S., Toshima T., Yokoyama M., Aida Y., Nishiwaki M., Kadowaki S., Narumi T., Honda Y. (2016). The role of macrophage transcription factor MafB in atherosclerotic plaque stability. Atherosclerosis.

[105] Franklin R.A., Liao W., Sarkar A., Kim M.V., Bivona M.R., Liu K., Pamer E.G., Li M.O. (2014). The cellular and molecular origin of tumor-associated macrophages. Science.

[106] Liu M., Tong Z., Ding C., Luo F., Wu S., Wu C., Albeituni S., He L., Hu X., Tieri D. (2020). Transcription factor c-Maf is a checkpoint that programs macrophages in lung cancer. J. Clin. Investig..

[107] Steingrímsson E., Copeland N.G., Jenkins N.A. (2004). Melanocytes and the microphthalmia transcription factor network. Annu. Rev. Genet..

[108] Kuiper R.P., Schepens M., Thijssen J., van Asseldonk M., van den Berg E., Bridge J., Schuuring E., Schoenmakers E.F., van Kessel A.G. (2003). Upregulation of the transcription factor TFEB in t (6; 11)(p21; q13)-positive renal cell carcinomas due to promoter substitution. Hum. Mol. Genet..

[109] Rehli M., Lichanska A., Cassady A.I., Ostrowski M.C., Hume D.A. (1999). TFEC is a macrophage-restricted member of the microphthalmia-TFE subfamily of basic helix-loop-helix leucine zipper transcription factors. J. Immunol..

[110] Van Vliet J., Crofts L.A., Quinlan K.G., Czolij R., Perkins A.C., Crossley M. (2006). Human KLF17 is a new member of the Sp/KLF family of transcription factors. Genomics.

[111] Katz J.P., Perreault N., Goldstein B.G., Lee C.S., Labosky P.A., Yang V.W., Kaestner K.H. (2002). The zinc-finger transcription factor Klf4 is required for terminal differentiation of goblet cells in the colon. Development.

[112] Petty A.J., Li A., Wang X., Dai R., Heyman B., Hsu D., Huang X., Yang Y. (2019). Hedgehog signaling promotes tumor-associated macrophage polarization to suppress intratumoral CD8+ T cell recruitment. J. Clin. Investig..

[113] Jain N., Shahal T., Gabrieli T., Gilat N., Torchinsky D., Michaeli Y., Vogel V., Ebenstein Y. (2019). Global Modulation in DNA Epigenetics during Pro-Inflammatory Macrophage Activation. bioRxiv.

[114] Bartel D.P. (2009). MicroRNAs: Target recognition and regulatory functions. Cell.

[115] Dekkers K.F., Neele A.E., Jukema J.W., Heijmans B.T., de Winther M.P. (2019). Human monocyte-to-macrophage differentiation involves highly localized gain and loss of DNA methylation at transcription factor binding sites. Epigenetics Chromatin.

[116] Wang X., Cao Q., Yu L., Shi H., Xue B., Shi H. (2016). Epigenetic regulation of macrophage polarization and inflammation by DNA methylation in obesity. JCI Insight.

[117] Zhang Y., Zhang M., Zhong M., Suo Q., Lv K. (2013). Expression profiles of miRNAs in polarized macrophages. Int. J. Mol. Med..

[118] Liu G., Abraham E. (2013). MicroRNAs in immune response and macrophage polarization. Arterioscler. Thromb. Vasc. Biol..

[119] Cai X., Yin Y., Li N., Zhu D., Zhang J., Zhang C.-Y., Zen K. (2012). Re-polarization of tumor-associated macrophages to pro-inflammatory M1 macrophages by microRNA-155. J. Mol. Cell Biol..

[120] Chen S., Yang J., Wei Y., Wei X. (2019). Epigenetic regulation of macrophages: From homeostasis maintenance to host defense. Cell. Mol. Immunol..

[121] Van den Bossche J., Neele A.E., Hoeksema M.A., De Winther M.P. (2014). Macrophage polarization: The epigenetic point of view. Curr. Opin. Lipidol..

[122] Schübeler D. (2015). Function and information content of DNA methylation. Nature.

[123] Bashkeel N., Perkins T.J., Kærn M., Lee J.M. (2019). Human gene expression variability and its dependence on methylation and aging. BMC Genom..

[124] Feinberg A.P. (2018). The key role of epigenetics in human disease prevention and mitigation. N. Engl. J. Med..

[125] Kelly A.D., Issa J.-P.J. (2017). The promise of epigenetic therapy: Reprogramming the cancer epigenome. Curr. Opin. Genet. Dev..

[126] Jurkowska R.Z., Jurkowski T.P., Jeltsch A. (2011). Structure and function of mammalian DNA methyltransferases. ChemBioChem.

[127] Rasmussen K.D., Helin K. (2016). Role of TET enzymes in DNA methylation, development, and cancer. Genes Dev..

[128] Rasmussen K.D., Helin K. (2015). TET1: An epigenetic guardian of lymphomagenesis. Nat. Immunol..

[129] Jambhekar A., Dhall A., Shi Y. (2019). Roles and regulation of histone methylation in animal development. Nat. Rev. Mol. Cell Biol..

[130] Liu F., Wang L., Perna F., Nimer S.D. (2016). Beyond transcription factors: How oncogenic signalling reshapes the epigenetic landscape. Nat. Rev. Cancer.

[131] Monticelli S. (2019). DNA (Hydroxy) Methylation in T Helper Lymphocytes. Trends Biochem. Sci..

[132] Yang X., Wang X., Liu D., Yu L., Xue B., Shi H. (2014). Epigenetic regulation of macrophage polarization by DNA methyltransferase 3b. Mol. Endocrinol..

[133] Cheng C., Huang C., Ma T.-T., Bian E.-B., He Y., Zhang L., Li J. (2014). SOCS1 hypermethylation mediated by DNMT1 is associated with lipopolysaccharide-induced inflammatory cytokines in macrophages. Toxicol. Lett..

[134] Yu J., Qiu Y., Yang J., Bian S., Chen G., Deng M., Kang H., Huang L. (2016). DNMT1-PPARγ pathway in macrophages regulates chronic inflammation and atherosclerosis development in mice. Sci. Rep..

[135] Pan W., Zhu S., Qu K., Meeth K., Cheng J., He K., Ma H., Liao Y., Wen X., Roden C. (2017). The DNA methylcytosine dioxygenase Tet2 sustains immunosuppressive function of tumor-infiltrating myeloid cells to promote melanoma progression. Immunity.

[136] Zhang Q., Zhao K., Shen Q., Han Y., Gu Y., Li X., Zhao D., Liu Y., Wang C., Zhang X. (2015). Tet2 is required to resolve inflammation by recruiting Hdac2 to specifically repress IL-6. Nature.

[137] Fan Z., Li J., Li P., Ye Q., Xu H., Wu X., Xu Y. (2017). Protein arginine methyltransferase 1 (PRMT1) represses MHC II transcription in macrophages by methylating CIITA. Sci. Rep..

[138] Tikhanovich I., Zhao J., Olson J., Adams A., Taylor R., Bridges B., Marshall L., Roberts B., Weinman S.A. (2017). Protein arginine methyltransferase 1 modulates innate immune responses through regulation of peroxisome proliferator-activated receptor γ-dependent macrophage differentiation. J. Biol. Chem..

[139] Li Y., Reddy M.A., Miao F., Shanmugam N., Yee J.-K., Hawkins D., Ren B., Natarajan R. (2008). Role of the histone H3 lysine 4 methyltransferase, SET7/9, in the regulation of NF-κB-dependent inflammatory genes relevance to diabetes and inflammation. J. Biol. Chem..

[140] Eames H., Saliba D., Krausgruber T., Lanfrancotti A., Ryzhakov G., Udalova I. (2012). KAP1/TRIM28: An inhibitor of IRF5 function in inflammatory macrophages. Immunobiology.

[141] Schliehe C., Flynn E.K., Vilagos B., Richson U., Swaminathan S., Bosnjak B., Bauer L., Kandasamy R.K., Griesshammer I.M., Kosack L. (2015). The methyltransferase Setdb2 mediates virus-induced susceptibility to bacterial superinfection. Nat. Immunol..

[142] Arts R.J., Blok B.A., van Crevel R., Joosten L.A., Aaby P., Benn C.S., Netea M.G. (2015). Vitamin A induces inhibitory histone methylation modifications and down-regulates trained immunity in human monocytes. J. Leukoc. Biol..

[143] Stender J.D., Pascual G., Liu W., Kaikkonen M.U., Do K., Spann N.J., Boutros M., Perrimon N., Rosenfeld M.G., Glass C.K. (2012). Control of proinflammatory gene programs by regulated trimethylation and demethylation of histone H4K20. Mol. Cell.

[144] Xu G., Liu G., Xiong S., Liu H., Chen X., Zheng B. (2015). The histone methyltransferase Smyd2 is a negative regulator of macrophage activation by suppressing interleukin 6 (IL-6) and tumor necrosis factor α (TNF-α) production. J. Biol. Chem..

[145] Xia M., Liu J., Wu X., Liu S., Li G., Han C., Song L., Li Z., Wang Q., Wang J. (2013). Histone methyltransferase Ash1l suppresses interleukin-6 production and inflammatory autoimmune diseases by inducing the ubiquitin-editing enzyme A20. Immunity.

[146] Kittan N.A., Allen R.M., Dhaliwal A., Cavassani K.A., Schaller M., Gallagher K.A., Carson IV W.F., Mukherjee S., Grembecka J., Cierpicki T. (2013). Cytokine induced phenotypic and epigenetic signatures are key to establishing specific macrophage phenotypes. PLoS ONE.

[147] Austenaa L., Barozzi I., Chronowska A., Termanini A., Ostuni R., Prosperini E., Stewart A.F., Testa G., Natoli G. (2012). The histone methyltransferase Wbp7 controls macrophage function through GPI glycolipid anchor synthesis. Immunity.

[148] Liu Y., Zhang Q., Ding Y., Li X., Zhao D., Zhao K., Guo Z., Cao X. (2015). Histone lysine methyltransferase Ezh1 promotes TLR-triggered inflammatory cytokine production by suppressing Tollip. J. Immunol..

[149] Nguyen K.D., Fentress S.J., Qiu Y., Yun K., Cox J.S., Chawla A. (2013). Circadian gene Bmal1 regulates diurnal oscillations of Ly6Chi inflammatory monocytes. Science.

[150] Neele A.E., Van den Bossche J., Hoeksema M.A., De Winther M.P. (2015). Epigenetic pathways in macrophages emerge as novel targets in atherosclerosis. Eur. J. Pharmacol..

[151] Chen X., El Gazzar M., Yoza B.K., McCall C.E. (2009). The NF-κB factor RelB and histone H3 lysine methyltransferase G9a directly interact to generate epigenetic silencing in endotoxin tolerance. J. Biol. Chem..

[152] Kapellos T.S., Iqbal A.J. (2016). Epigenetic control of macrophage polarisation and soluble mediator gene expression during inflammation. Mediat. Inflamm..

[153] Zhu Y., van Essen D., Saccani S. (2012). Cell-type-specific control of enhancer activity by H3K9 trimethylation. Mol. Cell.

[154] Kruidenier L., Chung C.-w., Cheng Z., Liddle J., Che K., Joberty G., Bantscheff M., Bountra C., Bridges A., Diallo H. (2012). A selective jumonji H3K27 demethylase inhibitor modulates the proinflammatory macrophage response. Nature.

[155] Ishii M., Wen H., Corsa C.A., Liu T., Coelho A.L., Allen R.M., Carson IV W.F., Cavassani K.A., Li X., Lukacs N.W. (2009). Epigenetic regulation of the alternatively activated macrophage phenotype. Blood J. Am. Soc. Hematol..

[156] Li X., Zhang Q., Shi Q., Liu Y., Zhao K., Shen Q., Shi Y., Liu X., Wang C., Li N. (2017). Demethylase Kdm6a epigenetically promotes IL-6 and IFN-β production in macrophages. J. Autoimmun..

[157] van Essen D., Zhu Y., Saccani S. (2010). A feed-forward circuit controlling inducible NF-κB target gene activation by promoter histone demethylation. Mol. Cell.

[158] Ka S.-O., Song M.-Y., Bae E.J., Park B.-H. (2015). Myeloid SIRT1 regulates macrophage infiltration and insulin sensitivity in mice fed a high-fat diet. J. Endocrinol..

[159] Shen Z., Ajmo J.M., Rogers C.Q., Liang X., Le L., Murr M.M., Peng Y., You M. (2009). Role of SIRT1 in regulation of LPS-or two ethanol metabolites-induced TNF-α production in cultured macrophage cell lines. Am. J. Physiol. -Gastrointest. Liver Physiol..

[160] Yoshizaki T., Schenk S., Imamura T., Babendure J.L., Sonoda N., Bae E.J., Oh D.Y., Lu M., Milne J.C., Westphal C. (2010). SIRT1 inhibits inflammatory pathways in macrophages and modulates insulin sensitivity. Am. J. Physiol. -Endocrinol. Metab..

[161] Li T., Garcia-Gomez A., Morante-Palacios O., Ciudad L., Özkaramehmet S., Van Dijck E., Rodríguez-Ubreva J., Vaquero A., Ballestar E. (2020). SIRT1/2 orchestrate acquisition of DNA methylation and loss of histone H3 activating marks to prevent premature activation of inflammatory genes in macrophages. Nucleic Acids Res..

[162] Mounce B.C., Mboko W.P., Kanack A.J., Tarakanova V.L. (2014). Primary macrophages rely on histone deacetylase 1 and 2 expression to induce type I interferon in response to gammaherpesvirus infection. J. Virol..

[163] Yao Z., Zhang Q., Li X., Zhao D., Liu Y., Zhao K., Liu Y., Wang C., Jiang M., Li N. (2014). Death domain-associated protein 6 (Daxx) selectively represses IL-6 transcription through histone deacetylase 1 (HDAC1)-mediated histone deacetylation in macrophages. J. Biol. Chem..

[164] Kong X., Fang M., Li P., Fang F., Xu Y. (2009). HDAC2 deacetylates class II transactivator and suppresses its activity in macrophages and smooth muscle cells. J. Mol. Cell. Cardiol..

[165] Chen X., Barozzi I., Termanini A., Prosperini E., Recchiuti A., Dalli J., Mietton F., Matteoli G., Hiebert S., Natoli G. (2012). Requirement for the histone deacetylase Hdac3 for the inflammatory gene expression program in macrophages. Proc. Natl. Acad. Sci. USA.

[166] Mullican S.E., Gaddis C.A., Alenghat T., Nair M.G., Giacomin P.R., Everett L.J., Feng D., Steger D.J., Schug J., Artis D. (2011). Histone deacetylase 3 is an epigenomic brake in macrophage alternative activation. Genes Dev..

[167] Wang B., Liu T.-y., Lai C.-H., Rao Y.-h., Choi M.-C., Chi J.-T., Dai J.-w., Rathmell J.C., Yao T.-P. (2014). Glycolysis-dependent histone deacetylase 4 degradation regulates inflammatory cytokine production. Mol. Biol. Cell.

[168] Yang Q., Wei J., Zhong L., Shi M., Zhou P., Zuo S., Wu K., Zhu M., Huang X., Yu Y. (2015). Cross talk between histone deacetylase 4 and STAT6 in the transcriptional regulation of arginase 1 during mouse dendritic cell differentiation. Mol. Cell. Biol..

[169] Poralla L., Stroh T., Erben U., Sittig M., Liebig S., Siegmund B., Glauben R. (2015). Histone deacetylase 5 regulates the inflammatory response of macrophages. J. Cell. Mol. Med..

[170] Yan B., Xie S., Liu Z., Ran J., Li Y., Wang J., Yang Y., Zhou J., Li D., Liu M. (2014). HDAC6 deacetylase activity is critical for lipopolysaccharide-induced activation of macrophages. PLoS ONE.

[171] Ariffin J.K., das Gupta K., Kapetanovic R., Iyer A., Reid R.C., Fairlie D.P., Sweet M.J. (2016). Histone deacetylase inhibitors promote mitochondrial reactive oxygen species production and bacterial clearance by human macrophages. Antimicrob. Agents Chemother..

[172] Villagra A., Cheng F., Wang H.-W., Suarez I., Glozak M., Maurin M., Nguyen D., Wright K.L., Atadja P.W., Bhalla K. (2009). The histone deacetylase HDAC11 regulates the expression of interleukin 10 and immune tolerance. Nat. Immunol..

[173] Stammler D., Eigenbrod T., Menz S., Frick J.S., Sweet M.J., Shakespear M.R., Jantsch J., Siegert I., Wölfle S., Langer J.D. (2015). Inhibition of Histone Deacetylases Permits Lipopolysaccharide-Mediated Secretion of Bioactive IL-1β via a Caspase-1–Independent Mechanism. J. Immunol..

[174] Nicodeme E., Jeffrey K.L., Schaefer U., Beinke S., Dewell S., Chung C.-w., Chandwani R., Marazzi I., Wilson P., Coste H. (2010). Suppression of inflammation by a synthetic histone mimic. Nature.

[175] Belkina A.C., Nikolajczyk B.S., Denis G.V. (2013). BET protein function is required for inflammation: Brd2 genetic disruption and BET inhibitor JQ1 impair mouse macrophage inflammatory responses. J. Immunol..

[176] Liu M., Zhou J., Chen Z., Cheng A.S.L. (2017). Understanding the epigenetic regulation of tumours and their microenvironments: Opportunities and problems for epigenetic therapy. J. Pathol..

[177] Audia J.E., Campbell R.M. (2016). Histone modifications and cancer. Cold Spring Harb. Perspect. Biol..

[178] Rodríguez-Ubreva J., Garcia-Gomez A., Ballestar E. (2017). Epigenetic mechanisms of myeloid differentiation in the tumor microenvironment. Curr. Opin. Pharmacol..

[179] Kzhyshkowska J., Rusch A., Wolf H., Dobner T. (2003). Regulation of transcription by the heterogeneous nuclear ribonucleoprotein E1B-AP5 is mediated by complex formation with the novel bromodomain-containing protein BRD7. Biochem. J..

[180] Dey A., Yang W., Gegonne A., Nishiyama A., Pan R., Yagi R., Grinberg A., Finkelman F.D., Pfeifer K., Zhu J. (2019). BRD4 directs hematopoietic stem cell development and modulates macrophage inflammatory responses. Embo J..

[181] Gatchalian J., Liao J., Maxwell M.B., Hargreaves D.C. (2020). Control of Stimulus-Dependent Responses in Macrophages by SWI/SNF Chromatin Remodeling Complexes. Trends Immunol..

[182] Ivashkiv L.B. (2013). Epigenetic regulation of macrophage polarization and function. Trends Immunol..

[183] Paul F., Arkin Y.a., Giladi A., Jaitin D.A., Kenigsberg E., Keren-Shaul H., Winter D., Lara-Astiaso D., Gury M., Weiner A. (2015). Transcriptional heterogeneity and lineage commitment in myeloid progenitors. Cell.

[184] Laurenti E., Göttgens B. (2018). From haematopoietic stem cells to complex differentiation landscapes. Nature.

[185] Lara-Astiaso D., Weiner A., Lorenzo-Vivas E., Zaretsky I., Jaitin D.A., David E., Keren-Shaul H., Mildner A., Winter D., Jung S. (2014). Chromatin state dynamics during blood formation. Science.

[186] Adams D., Altucci L., Antonarakis S.E., Ballesteros J., Beck S., Bird A., Bock C., Boehm B., Campo E., Caricasole A. (2012). BLUEPRINT to decode the epigenetic signature written in blood. Nat. Biotechnol..

[187] Chen X., Liu X., Zhang Y., Huai W., Zhou Q., Xu S., Chen X., Li N., Cao X. (2018). Methyltransferase Dot1l preferentially promotes innate IL-6 and IFN-β production by mediating H3K79me2/3 methylation in macrophages. Cell. Mol. Immunol..

[188] Jia Y., Han S., Li J., Wang H., Liu J., Li N., Yang X., Shi J., Han J., Li Y. (2017). IRF8 is the target of SIRT1 for the inflammation response in macrophages. Innate Immun..

[189] Czimmerer Z., Daniel B., Horvath A., Rückerl D., Nagy G., Kiss M., Peloquin M., Budai M.M., Cuaranta-Monroy I., Simandi Z. (2018). The transcription factor STAT6 mediates direct repression of inflammatory enhancers and limits activation of alternatively polarized macrophages. Immunity.

[190] Banerjee S., Halder K., Bose A., Bhattacharya P., Gupta G., Karmahapatra S., Das S., Chaudhuri S., Bhattacharyya Majumdar S., Majumdar S. (2011). TLR signaling-mediated differential histone modification at IL-10 and IL-12 promoter region leads to functional impairments in tumor-associated macrophages. Carcinogenesis.

[191] Joshi S., Singh A.R., Liu K.X., Pham T.V., Zulcic M., Skola D., Chun H.B., Glass C.K., Morales G.A., Garlich J.R. (2019). SF2523: Dual PI3K/BRD4 inhibitor blocks tumor immunosuppression and promotes adaptive immune responses in cancer. Mol. Cancer Ther..

[192] Chang Y.-C., Chen T.-C., Lee C.-T., Yang C.-Y., Wang H.-W., Wang C.-C., Hsieh S.-L. (2008). Epigenetic control of MHC class II expression in tumor-associated macrophages by decoy receptor 3. Blood.

[193] Boulding T., McCuaig R., Tan A., Hardy K., Wu F., Dunn J., Kalimutho M., Sutton C., Forwood J., Bert A. (2018). LSD1 activation promotes inducible EMT programs and modulates the tumour microenvironment in breast cancer. Sci. Rep..

[194] Groot A.E.d., Zarif J.C., Pienta K.J. (2017). Abstract 4005: HDAC inhibitors regulate M2 tumor-associated macrophage function through histone acetylation. Cancer Res..

[195] Tran K., Risingsong R.B., Royce D., Williams C.R., Sporn M.B., Pioli P.A., Gediya L.K., Njar V.C., Liby K.T. (2012). The combination of the histone deacetylase inhibitor vorinostat and synthetic triterpenoids reduces tumorigenesis in mouse models of cancer. Carcinogenesis.

[196] Osawa T., Tsuchida R., Muramatsu M., Shimamura T., Wang F., Suehiro J.-i., Kanki Y., Wada Y., Yuasa Y., Aburatani H. (2013). Inhibition of histone demethylase JMJD1A improves anti-angiogenic therapy and reduces tumor-associated macrophages. Cancer Res..

[197] Cooks T., Pateras I.S., Jenkins L.M., Patel K.M., Robles A.I., Morris J., Forshew T., Appella E., Gorgoulis V.G., Harris C.C. (2018). Mutant p53 cancers reprogram macrophages to tumor supporting macrophages via exosomal miR-1246. Nat. Commun..

[198] Poles W.A., Nishi E.E., de Oliveira M.B., Eugênio A.I., de Andrade T.A., Campos A.H.F., de Campos R.R., Vassallo J., Alves A.C., Neto C.S. (2019). Targeting the polarization of tumor-associated macrophages and modulating mir-155 expression might be a new approach to treat diffuse large B-cell lymphoma of the elderly. Cancer Immunol. Immunother..

[199] He M., Xu Z., Ding T., Kuang D.-M., Zheng L. (2009). MicroRNA-155 regulates inflammatory cytokine production in tumor-associated macrophages via targeting C/EBPβ. Cell. Mol. Immunol..

[200] Xu Z., Zhao L., Zhu L.-Y., He M., Zheng L., Wu Y. (2013). MicroRNA-17, 20a regulates the proangiogenic function of tumor-associated macrophages via targeting hypoxia-inducible factor 2α. PLoS ONE.

[201] Squadrito M.L., Pucci F., Magri L., Moi D., Gilfillan G.D., Ranghetti A., Casazza A., Mazzone M., Lyle R., Naldini L. (2012). miR-511-3p modulates genetic programs of tumor-associated macrophages. Cell Rep..

[202] Yang J., Zhang Z., Chen C., Liu Y., Si Q., Chuang T., Li N., Gomez-Cabrero A., Reisfeld R., Xiang R. (2014). MicroRNA-19a-3p inhibits breast cancer progression and metastasis by inducing macrophage polarization through downregulated expression of Fra-1 proto-oncogene. Oncogene.

[203] Guerriero J.L., Sotayo A., Ponichtera H.E., Castrillon J.A., Pourzia A.L., Schad S., Johnson S.F., Carrasco R.D., Lazo S., Bronson R.T. (2017). Class IIa HDAC inhibition reduces breast tumours and metastases through anti-tumour macrophages. Nature.

[204] Isayeva T., Brandwein-Gensler M., Somarathna M., Moore-Smith L.D., Lee T. (2017). Micro-RNA Profiling as a Predictor of Clinical Outcomes for Head and Neck Cancer Patients. Curr. Pharm. Des..

[205] Ying H., Kang Y., Zhang H., Zhao D., Xia J., Lu Z., Wang H., Xu F., Shi L. (2015). MiR-127 modulates macrophage polarization and promotes lung inflammation and injury by activating the JNK pathway. J. Immunol..

[206] Chaudhuri A.A., So A.Y.-L., Sinha N., Gibson W.S., Taganov K.D., O’Connell R.M., Baltimore D. (2011). MicroRNA-125b potentiates macrophage activation. J. Immunol..

[207] Cobos Jiménez V., Bradley E.J., Willemsen A.M., van Kampen A.H., Baas F., Kootstra N.A. (2013). Next-generation sequencing of microRNAs uncovers expression signatures in polarized macrophages. Physiol. Genom..

[208] Li D., Duan M., Feng Y., Geng L., Li X., Zhang W. (2016). MiR-146a modulates macrophage polarization in systemic juvenile idiopathic arthritis by targeting INHBA. Mol. Immunol..

[209] Movahedi K., Laoui D., Gysemans C., Baeten M., Stangé G., Van den Bossche J., Mack M., Pipeleers D., In’t Veld P., De Baetselier P. (2010). Different tumor microenvironments contain functionally distinct subsets of macrophages derived from Ly6C (high) monocytes. Cancer Res..

[210] Van den Bossche J., O’Neill L.A., Menon D. (2017). Macrophage immunometabolism: Where are we (going)?. Trends Immunol..

[211] Pavlou S., Lindsay J., Ingram R., Xu H., Chen M. (2018). Sustained high glucose exposure sensitizes macrophage responses to cytokine stimuli but reduces their phagocytic activity. BMC Immunol..

[212] Andrejeva G., Rathmell J.C. (2017). Similarities and distinctions of cancer and immune metabolism in inflammation and tumors. Cell Metab..

[213] Batista-Gonzalez A., Vidal R., Criollo A., Carreño L.J. (2020). New Insights on the Role of Lipid Metabolism in the Metabolic Reprogramming of Macrophages. Front. Immunol..

[214] Netea-Maier R.T., Smit J.W., Netea M.G. (2018). Metabolic changes in tumor cells and tumor-associated macrophages: A mutual relationship. Cancer Lett..

[215] Freemerman A.J., Johnson A.R., Sacks G.N., Milner J.J., Kirk E.L., Troester M.A., Macintyre A.N., Goraksha-Hicks P., Rathmell J.C., Makowski L. (2014). Metabolic reprogramming of macrophages glucose transporter 1 (GLUT1)-mediated glucose metabolism drives a proinflammatory phenotype. J. Biol. Chem..

[216] Rendra E., Riabov V., Mossel D.M., Sevastyanova T., Harmsen M.C., Kzhyshkowska J. (2019). Reactive oxygen species (ROS) in macrophage activation and function in diabetes. Immunobiology.

[217] Abais J.M., Xia M., Zhang Y., Boini K.M., Li P.-L. (2015). Redox regulation of NLRP3 inflammasomes: ROS as trigger or effector?. Antioxid. Redox Signal..

[218] Palsson-McDermott E.M., Curtis A.M., Goel G., Lauterbach M.A., Sheedy F.J., Gleeson L.E., van den Bosch M.W., Quinn S.R., Domingo-Fernandez R., Johnston D.G. (2015). Pyruvate kinase M2 regulates Hif-1α activity and IL-1β induction and is a critical determinant of the warburg effect in LPS-activated macrophages. Cell Metab..

[219] Xie M., Yu Y., Kang R., Zhu S., Yang L., Zeng L., Sun X., Yang M., Billiar T.R., Wang H. (2016). PKM2-dependent glycolysis promotes NLRP3 and AIM2 inflammasome activation. Nat. Commun..

[220] Tan Z., Xie N., Cui H., Moellering D.R., Abraham E., Thannickal V.J., Liu G. (2015). Pyruvate dehydrogenase kinase 1 participates in macrophage polarization via regulating glucose metabolism. J. Immunol..

[221] Min B.-K., Park S., Kang H.-J., Kim D.W., Ham H.J., Ha C.-M., Choi B.-J., Lee J.Y., Oh C.J., Yoo E.K. (2019). Pyruvate dehydrogenase kinase is a metabolic checkpoint for polarization of macrophages to the M1 phenotype. Front. Immunol..

[222] Semba H., Takeda N., Isagawa T., Sugiura Y., Honda K., Wake M., Miyazawa H., Yamaguchi Y., Miura M., Jenkins D.M. (2016). HIF-1α-PDK1 axis-induced active glycolysis plays an essential role in macrophage migratory capacity. Nat. Commun..

[223] Covarrubias A.J., Aksoylar H.I., Yu J., Snyder N.W., Worth A.J., Iyer S.S., Wang J., Ben-Sahra I., Byles V., Polynne-Stapornkul T. (2016). Akt-mTORC1 signaling regulates Acly to integrate metabolic input to control of macrophage activation. elife.

[224] Huang S.C.-C., Smith A.M., Everts B., Colonna M., Pearce E.L., Schilling J.D., Pearce E.J. (2016). mTORC2-IRF4 mediated metabolic reprograming is essential for macrophage alternative activation. Immunity.

[225] Wang F., Zhang S., Vuckovic I., Jeon R., Lerman A., Folmes C.D., Dzeja P.P., Herrmann J. (2018). Glycolytic stimulation is not a requirement for M2 macrophage differentiation. Cell Metab..

[226] Kang Y.E., Kim H.J., Shong M. (2019). Regulation of Systemic Glucose Homeostasis by T Helper Type 2 Cytokines. Diabetes Metab. J..

[227] Geeraerts X., Bolli E., Fendt S.-M., Van Ginderachter J.A. (2017). Macrophage metabolism as therapeutic target for cancer, atherosclerosis, and obesity. Front. Immunol..

[228] Tan H.-Y., Wang N., Li S., Hong M., Wang X., Feng Y. (2016). The reactive oxygen species in macrophage polarization: Reflecting its dual role in progression and treatment of human diseases. Oxidative Med. Cell. Longev..

[229] Baardman J., Verberk S.G., Prange K.H., van Weeghel M., van der Velden S., Ryan D.G., Wüst R.C., Neele A.E., Speijer D., Denis S.W. (2018). A defective pentose phosphate pathway reduces inflammatory macrophage responses during hypercholesterolemia. Cell Rep..

[230] Williams N.C., O’Neill L.A. (2018). A role for the Krebs cycle intermediate citrate in metabolic reprogramming in innate immunity and inflammation. Front. Immunol..

[231] Infantino V., Convertini P., Cucci L., Panaro M.A., Di Noia M.A., Calvello R., Palmieri F., Iacobazzi V. (2011). The mitochondrial citrate carrier: A new player in inflammation. Biochem. J..

[232] Tannahill G., Curtis A., Adamik J., Palsson-McDermott E., McGettrick A., Goel G., Frezza C., Bernard N., Kelly B., Foley N. (2013). Succinate is an inflammatory signal that induces IL-1β through HIF-1α. Nature.

[233] Van den Bossche J., Baardman J., Otto N.A., van der Velden S., Neele A.E., van den Berg S.M., Luque-Martin R., Chen H.-J., Boshuizen M.C., Ahmed M. (2016). Mitochondrial dysfunction prevents repolarization of inflammatory macrophages. Cell Rep..

[234] Torres-Castro I., Arroyo-Camarena Ú.D., Martínez-Reyes C.P., Gómez-Arauz A.Y., Dueñas-Andrade Y., Hernández-Ruiz J., Béjar Y.L., Zaga-Clavellina V., Morales-Montor J., Terrazas L.I. (2016). Human monocytes and macrophages undergo M1-type inflammatory polarization in response to high levels of glucose. Immunol. Lett..

[235] Moganti K., Li F., Schmuttermaier C., Riemann S., Klüter H., Gratchev A., Harmsen M.C., Kzhyshkowska J. (2017). Hyperglycemia induces mixed M1/M2 cytokine profile in primary human monocyte-derived macrophages. Immunobiology.

[236] Mossel D.M., Moganti K., Riabov V., Weiß C., Kopf S., Cordero J., Dobreva G., Rots M., Klüter H., Harmsen M.C. Epigenetic regulation of S100A9 and S100A12 expression in monocyte- macrophage system in hyperglycemic conditions. Front. Immunol..

[237] Voronov E., Apte R.N. (2017). Targeting the tumor microenvironment by intervention in interleukin-1 biology. Curr. Pharm. Des..

[238] Boraschi D., Apte R.N., Martin M.U. (2019). One hits (almost) all. Nat. Immunol..

[239] Shabani F., Farasat A., Mahdavi M., Gheibi N. (2018). Calprotectin (S100A8/S100A9): A key protein between inflammation and cancer. Inflamm. Res..

[240] Huang S.C.-C., Everts B., Ivanova Y., O’sullivan D., Nascimento M., Smith A.M., Beatty W., Love-Gregory L., Lam W.Y., O’neill C.M. (2014). Cell-intrinsic lysosomal lipolysis is essential for alternative activation of macrophages. Nat. Immunol..

[241] Moon J.-S., Nakahira K., Chung K.-P., DeNicola G.M., Koo M.J., Pabón M.A., Rooney K.T., Yoon J.-H., Ryter S.W., Stout-Delgado H. (2016). NOX4-dependent fatty acid oxidation promotes NLRP3 inflammasome activation in macrophages. Nat. Med..

[242] Wen H., Gris D., Lei Y., Jha S., Zhang L., Huang M.T.-H., Brickey W.J., Ting J.P. (2011). Fatty acid–induced NLRP3-ASC inflammasome activation interferes with insulin signaling. Nat. Immunol..

[243] Seth P., Csizmadia E., Hedblom A., Vuerich M., Xie H., Li M., Longhi M.S., Wegiel B. (2017). Deletion of lactate dehydrogenase-A in myeloid cells triggers antitumor immunity. Cancer Res..

[244] Lin S., Sun L., Lyu X., Ai X., Du D., Su N., Li H., Zhang L., Yu J., Yuan S. (2017). Lactate-activated macrophages induced aerobic glycolysis and epithelial-mesenchymal transition in breast cancer by regulation of CCL5-CCR5 axis: A positive metabolic feedback loop. Oncotarget.

[245] Penny H.L., Sieow J.L., Adriani G., Yeap W.H., See Chi Ee P., San Luis B., Lee B., Lee T., Mak S.Y., Ho Y.S. (2016). Warburg metabolism in tumor-conditioned macrophages promotes metastasis in human pancreatic ductal adenocarcinoma. OncoImmunology.

[246] Liu D., Chang C., Lu N., Wang X., Lu Q., Ren X., Ren P., Zhao D., Wang L., Zhu Y. (2017). Comprehensive proteomics analysis reveals metabolic reprogramming of tumor-associated macrophages stimulated by the tumor microenvironment. J. Proteome Res..

[247] De-Brito N.M., Duncan-Moretti J., da-Costa H.C., Saldanha-Gama R., Paula-Neto H.A., Dorighello G.G., Simões R.L., Barja-Fidalgo C. (2020). Aerobic glycolysis is a metabolic requirement to maintain the M2-like polarization of tumor-associated macrophages. Biochim. Biophys. Acta (Bba)-Mol. Cell Res..

[248] Arts R.J., Plantinga T.S., Tuit S., Ulas T., Heinhuis B., Tesselaar M., Sloot Y., Adema G.J., Joosten L.A., Smit J.W. (2016). Transcriptional and metabolic reprogramming induce an inflammatory phenotype in non-medullary thyroid carcinoma-induced macrophages. OncoImmunology.

[249] Wenes M., Shang M., Di Matteo M., Goveia J., Martín-Pérez R., Serneels J., Prenen H., Ghesquière B., Carmeliet P., Mazzone M. (2016). Macrophage metabolism controls tumor blood vessel morphogenesis and metastasis. Cell Metab..

[250] Carmona-Fontaine C., Deforet M., Akkari L., Thompson C.B., Joyce J.A., Xavier J.B. (2017). Metabolic origins of spatial organization in the tumor microenvironment. Proc. Natl. Acad. Sci. USA.

[251] Colegio O.R., Chu N.-Q., Szabo A.L., Chu T., Rhebergen A.M., Jairam V., Cyrus N., Brokowski C.E., Eisenbarth S.C., Phillips G.M. (2014). Functional polarization of tumour-associated macrophages by tumour-derived lactic acid. Nature.

[252] Huber R., Meier B., Otsuka A., Fenini G., Satoh T., Gehrke S., Widmer D., Levesque M.P., Mangana J., Kerl K. (2016). Tumour hypoxia promotes melanoma growth and metastasis via High Mobility Group Box-1 and M2-like macrophages. Sci. Rep..

[253] Zhang J., Li H., Wu Q., Chen Y., Deng Y., Yang Z., Zhang L., Liu B. (2019). Tumoral NOX4 recruits M2 tumor-associated macrophages via ROS/PI3K signaling-dependent various cytokine production to promote NSCLC growth. Redox Biol..

[254] Guo X., Xue H., Shao Q., Wang J., Guo X., Chen X., Zhang J., Xu S., Li T., Zhang P. (2016). Hypoxia promotes glioma-associated macrophage infiltration via periostin and subsequent M2 polarization by upregulating TGF-beta and M-CSFR. Oncotarget.

[255] Park J.E., Dutta B., Tse S.W., Gupta N., Tan C.F., Low J.K., Yeoh K.W., Kon O.L., Tam J.P., Sze S.K. (2019). Hypoxia-induced tumor exosomes promote M2-like macrophage polarization of infiltrating myeloid cells and microRNA-mediated metabolic shift. Oncogene.

[256] Palmieri E.M., Menga A., Martín-Pérez R., Quinto A., Riera-Domingo C., De Tullio G., Hooper D.C., Lamers W.H., Ghesquière B., McVicar D.W. (2017). Pharmacologic or genetic targeting of glutamine synthetase skews macrophages toward an M1-like phenotype and inhibits tumor metastasis. Cell Rep..

[257] Wu H., Han Y., Sillke Y.R., Deng H., Siddiqui S., Treese C., Schmidt F., Friedrich M., Keye J., Wan J. (2019). Lipid droplet-dependent fatty acid metabolism controls the immune suppressive phenotype of tumor-associated macrophages. Embo Mol. Med..

[258] Zhang Y., Sun Y., Rao E., Yan F., Li Q., Zhang Y., Silverstein K.A., Liu S., Sauter E., Cleary M.P. (2014). Fatty acid-binding protein E-FABP restricts tumor growth by promoting IFN-β responses in tumor-associated macrophages. Cancer Res..

[259] Daurkin I., Eruslanov E., Stoffs T., Perrin G.Q., Algood C., Gilbert S.M., Rosser C.J., Su L.-M., Vieweg J., Kusmartsev S. (2011). Tumor-associated macrophages mediate immunosuppression in the renal cancer microenvironment by activating the 15-lipoxygenase-2 pathway. Cancer Res..

[260] Weiss J.M., Davies L.C., Karwan M., Ileva L., Ozaki M.K., Cheng R.Y., Ridnour L.A., Annunziata C.M., Wink D.A., McVicar D.W. (2018). Itaconic acid mediates crosstalk between macrophage metabolism and peritoneal tumors. J. Clin. Investig..

[261] Chen F., Chen J., Yang L., Liu J., Zhang X., Zhang Y., Tu Q., Yin D., Lin D., Wong P.-P. (2019). Extracellular vesicle-packaged HIF-1α-stabilizing lncRNA from tumour-associated macrophages regulates aerobic glycolysis of breast cancer cells. Nat. Cell Biol..

[262] Jeong H., Kim S., Hong B.-J., Lee C.-J., Kim Y.-E., Bok S., Oh J.-M., Gwak S.-H., Yoo M.Y., Lee M.S. (2019). Tumor-associated macrophages enhance tumor hypoxia and aerobic glycolysis. Cancer Res..

[263] Riboldi E., Porta C., Morlacchi S., Viola A., Mantovani A., Sica A. (2013). Hypoxia-mediated regulation of macrophage functions in pathophysiology. Int. Immunol..

[264] Burke B., Giannoudis A., Corke K.P., Gill D., Wells M., Ziegler-Heitbrock L., Lewis C.E. (2003). Hypoxia-induced gene expression in human macrophages: Implications for ischemic tissues and hypoxia-regulated gene therapy. Am. J. Pathol..

[265] Henze A.-T., Mazzone M. (2016). The impact of hypoxia on tumor-associated macrophages. J. Clin. Investig..

[266] Fang H.-Y., Hughes R., Murdoch C., Coffelt S.B., Biswas S.K., Harris A.L., Johnson R.S., Imityaz H.Z., Simon M.C., Fredlund E. (2009). Hypoxia-inducible factors 1 and 2 are important transcriptional effectors in primary macrophages experiencing hypoxia. Bloodthe J. Am. Soc. Hematol..

[267] Kumar V., Gabrilovich D.I. (2014). Hypoxia-inducible factors in regulation of immune responses in tumour microenvironment. Immunology.

[268] Mi Z., Rapisarda A., Taylor L., Brooks A., Creighton-Gutteridge M., Melillo G., Varesio L. (2008). Synergystic induction of HIF-1α transcriptional activity by hypoxia and lipopolysaccharide in macrophages. Cell Cycle.

[269] Tafani M., Sansone L., Limana F., Arcangeli T., De Santis E., Polese M., Fini M., Russo M.A. (2016). The interplay of reactive oxygen species, hypoxia, inflammation, and sirtuins in cancer initiation and progression. Oxidative Med. Cell. Longev..

[270] Zhang Y., Choksi S., Chen K., Pobezinskaya Y., Linnoila I., Liu Z.-G. (2013). ROS play a critical role in the differentiation of alternatively activated macrophages and the occurrence of tumor-associated macrophages. Cell Res..

[271] Roux C., Jafari S.M., Shinde R., Duncan G., Cescon D.W., Silvester J., Chu M.F., Hodgson K., Berger T., Wakeham A. (2019). Reactive oxygen species modulate macrophage immunosuppressive phenotype through the up-regulation of PD-L1. Proc. Natl. Acad. Sci. USA.

[272] Ke X., Chen C., Song Y., Cai Q., Li J., Tang Y., Han X., Qu W., Chen A., Wang H. (2019). Hypoxia modifies the polarization of macrophages and their inflammatory microenvironment, and inhibits malignant behavior in cancer cells. Oncol. Lett..

[273] Guan X.L., Schmidt A., Bumann D., Rosas-Ballina M. (2020). Classical activation of macrophages leads to lipid droplet formation without de novo fatty acid synthesis. Front. Immunol..

[274] Choi J., Stradmann-Bellinghausen B., Yakubov E., Savaskan N.E., Régnier-Vigouroux A. (2015). Glioblastoma cells induce differential glutamatergic gene expressions in human tumor-associated microglia/macrophages and monocyte-derived macrophages. Cancer Biol. Ther..

[275] EmmaWard R., Branca J.V., Noakes D., Morucci G., Thyer L. (2014). Clinical experience of cancer immunotherapy integrated with oleic acid complexed with de-glycosylated vitamin d binding protein. Am. J. Immunol..

[276] Nonaka K., Onizuka S., Ishibashi H., Uto Y., Hori H., Nakayama T., Matsuura N., Kanematsu T., Fujioka H. (2012). Vitamin D binding protein-macrophage activating factor inhibits HCC in SCID mice. J. Surg. Res..

[277] Oh M.-H., Sun I.-H., Zhao L., Leone R.D., Sun I.-M., Xu W., Collins S.L., Tam A.J., Blosser R.L., Patel C.H. (2020). Targeting glutamine metabolism enhances tumor specific immunity by modulating suppressive myeloid cells. J. Clin. Investig..

